# CD44-targeted virus-mimicking nanomedicine eliminates cancer stem cells and mitigates chemoresistance in head and neck squamous cell carcinoma

**DOI:** 10.1016/j.mtbio.2025.101721

**Published:** 2025-03-29

**Authors:** Yiwen Chen, Zhen Qin, Yujia Wang, Baoxin Gu, Jing Wang, Yunfei Zheng, Yuting Niu, Lingfei Jia

**Affiliations:** a Department of Oral and Maxillofacial Surgery, Peking University School and Hospital of Stomatology, Beijing, 100081, PR China; b National Center for Stomatology & National Clinical Research Center for Oral Diseases & National Engineering Research Center of Oral Biomaterials and Digital Medical Devices, Beijing, 100081, PR China; c State Key Laboratory of Natural and Biomimetic Drugs, Peking University, Beijing, 100091, PR China; d Department of Orthodontics, Peking University School and Hospital of Stomatology, Beijing, 100081, PR China; e Central Laboratory, Peking University School and Hospital of Stomatology, Beijing, 100081, PR China; f Beijing Advanced Center of Cellular Homeostasis and Aging-Related Diseases, Institute of Advanced Clinical Medicine, Peking University, Beijing 100091, PR China

**Keywords:** Cancer stem cells, CD44 targeting, Cisplatin resistance, Head and neck squamous cell carcinoma, PTC209, Virus-mimicking nanoparticles

## Abstract

Cancer stem cells (CSCs) play critical roles in tumor growth, metastasis, and chemoresistance. Although several small-molecule inhibitors designed to inhibit CSCs have been investigated in clinical trials, their inadequate tumor targeting and potential off-target side effects have led to poor outcomes. A CD44-targeted virus-mimicking nanomedicine encapsulating the BMI1 inhibitor PTC209 (PTC209@VNP-HA) was designed to treat head and neck squamous cell carcinoma (HNSCC). We used a dendritic mesoporous silica nanoparticle (MSN) as the core for virus-mimicking nanoparticle (VNP) formation after adding shell particles to the MSN surface. The VNP surface was then modified with hyaluronic acid (HA), and PTC209 was adsorbed by mesopores to form PTC209@VNP-HA. In this system, HA is used to target CD44^+^ CSCs. The rough surface of VNP-HA provided better drug delivery efficiency than smooth nanoparticles modified with HA. VNP-HA enhanced the cancer inhibitory effect of PTC209 12-fold compared to the administration of free PTC209, leading to significantly higher bioavailability of PTC209. Both *in vitro* and *in vivo* assays showed that PTC209@VNP-HA inhibited cancer stemness, proliferation, and metastasis in HNSCC. Mechanistically, this inhibitory effect is closely associated with DNA damage/apoptosis signaling. Using a series of preclinical models in murine systems, we confirmed that PTC209@VNP-HA eliminated BMI1^+^ CSCs, and greatly inhibited the proliferation and metastasis of HNSCC when combined with cisplatin. This study investigated PTC209@VNP-HA as a novel and potentially transformative HNSCC treatment option that eliminates CSCs, prevents metastasis, and overcomes cisplatin resistance.

## Highlights

• A novel virus-mimicking nanomedicine targeting cancer stem cells was constructed.
•The rough viral-mimicking design improves the delivery efficiency of PTC209 relative to the smooth design.
•The nanodelivery system boosts anti-cancer efficacy 12 times by effectively delivering PTC209 to cancer stem cells.
•Combination of nanomedicine and cisplatin mitigates chemoresistance and inhibits metastasis in head and neck squamous cell carcinoma.

## Graphical abstract

**Figure:**
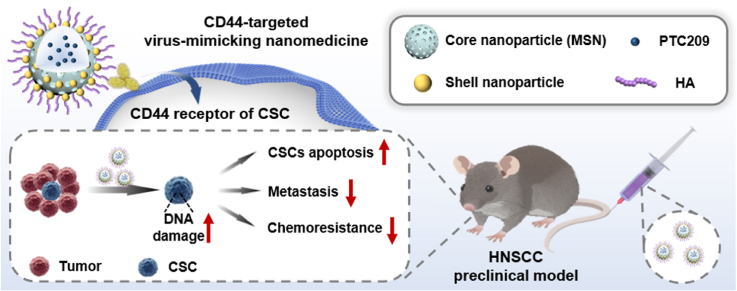

## 1 Introduction

Head and neck squamous cell carcinoma (HNSCC) derives from the oral cavity, oropharynx, larynx, and hypopharynx, and exhibits high invasiveness and a propensity for cervical lymph node metastasis [1]. Despite significant therapeutic advancements, the 5-year survival rate for HNSCC patients remains low [2]. Cancer stem cells (CSCs) play a pivotal role in driving tumor growth, metastasis, recurrence, and drug resistance in HNSCC [3], highlighting the need for CSC-targeted therapies. BMI1, a crucial component of polycomb repressive complex 1 (PRC1), which facilitates gene silencing, functions as a key self-renewal factor for CSCs. Our recent *in vivo* lineage tracing study suggested that BMI1 inhibition could be a promising strategy for CSC elimination and HNSCC treatment [4].

PTC209, a potent and selective BMI1 inhibitor, effectively reduces BMI1 expression and demonstrates robust anti-tumor activity against various cancer cell types, including biliary tract cancer, acute myeloid leukemia, glioblastoma, myeloma, squamous cell carcinoma, and colon, breast, and lung cancers [[4], [5], [6], [7], [8], [9], [10]]. However, the degradation and denaturation of PTC209 in the bloodstream can lead to inefficient delivery, creating substantial challenges for its clinical application [11]. Furthermore, BMI1 plays a vital role in the self-renewal and maintenance of normal stem cells in the prostate, intestine, lung epithelium, and skin [[12], [13], [14]]. Therefore, achieving the selective inhibition of BMI1 in tumor tissues while preserving its functionality in normal tissues is an important goal in cancer research.

Nanoparticle systems have been widely utilized as carriers for anti-tumor drugs, bolstering their anti-tumor efficacy while minimizing adverse effects [15]. Nanoparticles of specific sizes tend to aggregate within targeted tumor tissues due to enhanced permeability and retention (EPR) effects caused by abnormal vascular leakage and impaired lymphatic drainage within the tumor region [16,17]. However, reliance solely on EPR-mediated passive targeting is inadequate for achieving highly effective CSC-specific targeted therapy [18]. The concentration of therapeutic agents on CSCs can be significantly enhanced via active ligand–receptor interactions by decorating the nanoparticle surface with CSC-targeting ligands [19]. Hyaluronic acid (HA) has been used as a targeting ligand in cancer therapy because it is natural, biocompatible, and biodegradable. The CD44 receptor, a protein commonly found in high levels on the surfaces of CSCs across various tumor types, makes HA an excellent choice for targeting and binding CSCs [20,21].

Numerous synthetic vectors, including nanoparticles, are inefficient for cellular delivery, whereas viruses are more easily tailored for this task due to their high cell infection rates [13,22]. We previously synthesized a virus-mimicking nanoparticle and systematically studied its cellular delivery efficiency, including its cargo molecule binding capacity and subsequent cellular uptake [23]. However, this nanoparticle performs only limited biological functions; it cannot act as a small molecule inhibitors carrier or connect to molecules with targeted properties. Thus, it remains challenging to develop nanoparticle-based carrier systems with high delivery efficiency.

To address these challenges, we designed an innovative CD44-targeted virus-mimicking nanomedicine (PTC209@VNP-HA) that enhances the local delivery efficacy of the CSC-targeted drug PTC209. Mesoporous silica nanoparticles (MSNs) have an extensive surface area and superior drug loading capacity compared to other carriers, and are therefore used as drug containers in many systems [24]. The proposed silica VNP combines MSN core particles (110 nm) with smaller shell particles (10 nm), roughening the surface of the core. We further functionalized the nanoparticle surface with HA to improve its CSC targeting ability and biological tissue compatibility. Then, PTC209 was adsorbed onto the mesoporous structure of the MSN core to generate PTC209@VNP-HA (Schematic 1a). The therapeutic efficacy of PTC209@VNP-HA in HNSCC was validated in subcutaneous, orthotopic, metastatic, and genetic CSC lineage-tracing spontaneous HNSCC mouse models. The resulting nanomedicine represents an effective strategy for eliminating CSCs, inhibiting metastasis, and reversing chemoresistance in HNSCC (Schematic 1b).

**Schematic 1:**
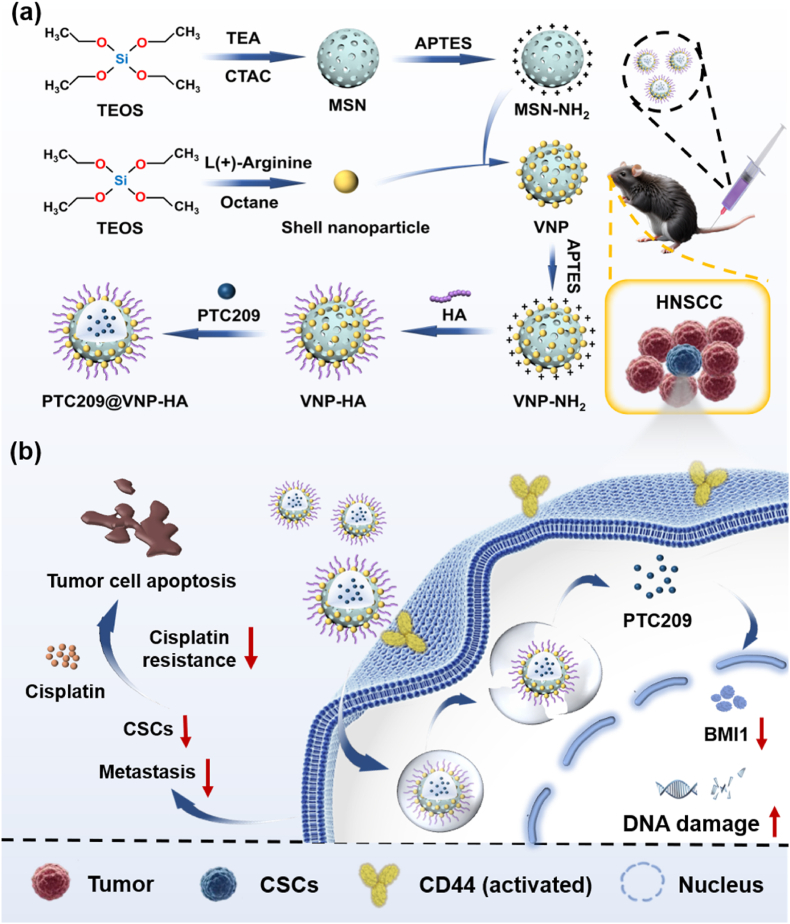
**Schematic diagram of the preparation and biological mechanism of the novel nanomedicine.** (a) A mesoporous silica nanoparticle (MSN) was fabricated, and its surface was modified by amination with 3-aminopropyltriethoxysilane (APTES) to form MSN-NH_2_. It was conjugated to shell particles to form the virus-mimicking nanoparticle (VNP). Then VNP further aminated to form VNP-NH_2_. The nanoparticle surface was modified with hyaluronic acid (HA) to synthesize the VNP-HA nanoplatform, which is used to carry the BMI1 inhibitor PTC209 as a nanodrug (PTC209@VNP-HA). (b) Working principles of PTC209@VNP-HA in targeted chemotherapy *in vivo*. After targeting cancer stem cells (CSCs), the PTC209@VNP-HA nanoplatform releases PTC209, which inhibits BMI1 and causes DNA damage, thereby reducing tumor stemness and overcoming chemoresistance. CTAC, cetyltrimethylammonium chloride; TEA, triethanolamine.

## 2 Materials and methods

### 2.1 Synthesis of MSNs

Dendritic MSNs were synthesized using a straightforward single-pot biphasic layering technique. The silicon source was tetraethyl orthosilicate (TEOS; Shanghai, China), and the structural template was composed of cetyltrimethylammonium chloride (CTAC, Aladdin Biochemical Technology, Shanghai, China). Cyclohexane (Macklin Biochemical, Shanghai, China) was used as an emulsifier to facilitate the formation of a stable biphasic system. Triethanolamine (Aladdin Biochemical Technology) was used as both a catalyst and pH modifier. A round-bottomed flask was loaded with 0.36 g triethanolamine, 25 wt% CTAC solution, and 72 mL of deionized water, for a total volume of 48 mL. The mixture was stirred at 60 °C for at least 1 h to ensure the formation of micelles, which acted as scaffolds for the MSN structure. Next, 40 mL of a 20 v/v% solution of TEOS in cyclohexane was added to the flask. The reaction mixture was stirred continuously at 60 °C for 24 h, allowing TEOS to condense around the micelles and gradually build up the MSN framework. After the reaction, the milky aqueous phase containing MSNs was separated using a pear-shaped separatory funnel and centrifuged at 20,000×*g* for 60 min to pellet the nanoparticles. The pellet was then rigorously extracted with acidic methanol (prepared by mixing 37 % HCl with methanol in a 1:10 ratio) for eight 6 h cycles at 60 °C to ensure complete removal of the CTAC template. The prepared MSNs were stored in ethanol at room temperature until further use.

### 2.2 Synthesis of MSN-NH_2_

Aminosilanes were grafted onto MSNs to change the MSN surface from hydroxyl-negative to amino-positive. A stirrer was heated to 145 °C while MSNs (250 mg) were added to a round-bottomed flask containing 150 mL of xylene, and 2.5 mL of 3-aminopropyltriethoxysilane (APTES, Sigma-Aldrich, St. Louis, MO, USA) was added dropwise. The flask was connected to a serpentine distillation tube reflux device, and the mixture was stirred at 145 °C for 12 h using the heated stirrer. At the end of the reaction, the MSNs were centrifuged at 20,000×*g* for 30 min and then washed with ethanol three times to remove xylene and residual APTES, yielding MSN-NH_2_. The product was stored in ethanol until further use.

### 2.3 Synthesis of VNPs

L-arginine (87 mg) was dissolved in deionized water (69.5 mL) with octane (5.23 mL). TEOS (0.5 mL) was added, and the mixture was sonicated and stirred at 60 °C for 3 h to form shell particles. Amino-modified core (MSN-NH_2_) particles (100 mg) were suspended in carbonate buffer (50 mL, pH 9.5, Ct [CO_3_^2−^] = 50 mmol/L). The shell particle suspension (16 mL) was added dropwise to the MSN-NH_2_ solution, and the reaction proceeded for 8 h at room temperature to synthesize VNPs. The VNPs were washed with ethanol and stored in ethanol at room temperature until further use.

### 2.4 Synthesis of VNP-NH_2_

Aminosilanes were grafted onto VNP shell particles to create a biologically active surface on which to graft HA. A stirrer was heated to 145 °C while VNPs (250 mg) were added to 150 mL of xylene. Then, 2.5 mL of APTES were added dropwise and the mixture was stirred at 145 °C for 12 h using a serpentine rectifier with the heated stirrer. VNP-NH_2_ was centrifuged at 20,000×*g* for 30 min and washed with ethanol three times to remove xylene and residual APTES, yielding MSN-NH_2_. The product was stored in ethanol until further use.

### 2.5 Synthesis of VNP-HA

The carboxyl groups of HA (32 mg) were activated using 1-(3-dimethylaminopropyl)-3-ethylcarbodiimide hydrochloride (EDC, 12 mg) as a catalyst in phosphate-buffered saline (PBS, pH 7.4, 25 mL) at room temperature for a duration of 0.5 h. N-hydroxysuccinimide (NHS, 14 mg) was added and the mixture was stirred continuously for another 0.5 h. Then, VNP-NH_2_ (100 mg) in PBS (pH = 7.4, 25 mL) was added, and the NHS-activated HA was further reacted for 3 h. The VNP-HA product was washed three times with deionized water (20 mL per wash) and ethanol (20 mL per wash) to remove unreacted chemicals. The final targeting delivery system, VNP-HA, was maintained under wet conditions at 4 °C.

### 2.6 Synthesis of PTC@VNP-HA and calculation of the PTC209 load

PTC209 (MedChemExpress, Monmouth Junction, NJ, USA) was loaded into VNP-HA via rotary evaporation by mixing 2.5 mg PTC209, 7.5 mg VNP-HA, and 0.5 mL dimethylformamide (DMF). Complete DMF evaporation under nitrogen yielded a dry PTC209@VNP-HA powder. Scanning electron microscopy (SEM; 5 kV; GeminiSEM 500, Zeiss, Oberkochen, Germany) showed that PTC209@VNP-HA sample has no crystallization of free PTC209, confirming uniform encapsulation. Gravimetric analysis revealed a loading efficiency of 0.25 mg PTC209 per mg PTC209@VNP-HA.

### 2.7 Characterization of nanoparticles

Transmission electron microscopy (TEM; 100 kV, JEOL 1400, JEOL, Tokyo, Japan) measurements were conducted by dispersing a small sample in ethanol and transferring it onto a copper grid. Images of PTC209@MSN-HA and PTC209@VNP-HA samples were obtained through high-resolution TEM (HRTEM) and energy-dispersive X-ray (EDX) spectroscopy. SEM provided insights into the surface morphology and encapsulation effects of the nanoparticles. Thermogravimetric analysis (TGA) was conducted to evaluate the thermal stability and composition of the samples using a thermogravimetric analyzer–digital scanning calorimeter (Q600, TA Instruments, New Castle, DE, USA). Measurements of ζ-potential and dynamic light scattering were conducted using a Zetasizer Pro analysis system (Malvern Panalytical, Malvern, UK) to determine the surface charge and particle size distribution, respectively. Nitrogen adsorption–desorption measurements were conducted at −196 °C using an ASAP 2020 analyzer (Micromeritics, Norcross, GA, USA) to obtain porosity data, including specific surface area, pore size distribution, and total pore volume. Fourier-transform infrared spectroscopy (FTIR) analysis of MSN-NH_2_, MSN-HA, VNP-NH_2_, VNP-HA, and HA samples (Nicolet-6700, Thermo Fisher Scientific, Waltham, MA, USA) was conducted to confirm the successful attachment of HA to the nanoparticles by identifying specific chemical bonds and functional groups.

### 2.8 Release profile of PTC209 by PTC209@VNP-HA

The *in vitro* release profile of PTC209 from PTC209@VNP-HA was assessed through a dialysis method conducted in PBS with pH values of 7.4, 6.5, and 5.5. An aliquot of PTC209@VNP-HA dispersion was transferred into a dialysis membrane bag (Sigma-Aldrich) with a molecular weight cutoff of 2000 Da, to ensure that only smaller molecules could freely diffuse out of the bag while larger nanoparticles remained confined. The dialysis bags were placed in a shaker maintained at a constant temperature of 37 °C and agitated at 100 rpm to facilitate the diffusion process. We collected 1.5 mL of the released medium while simultaneously adding an equal volume of PBS to the dialysis system to maintain a constant volume and ensure continuous diffusion. Each sample was centrifuged at 20,000×*g* for 10 min to separate the supernatant, which contained the solubilized PTC209. From the centrifuged supernatant, 1 mL was removed and mixed with 0.5 mL of acetonitrile (Macklin Biochemical), a solvent that enhances the solubility and spectral properties of PTC209. The resulting mixture was analyzed using an ultraviolet–visible light spectrophotometer (Cary 60, Agilent Technologies, Santa Clara, CA, USA) to measure the absorbance of the solution at a wavelength of 235 nm. The concentration of PTC209 in each sample was determined by comparing the measured absorbance values to a calibration curve. These concentrations were then plotted against time to generate a release curve.

### 2.9 Binding specificity of VNP-HA to CD44 protein

A biological layer interferometry (BLI) assay was performed using the Octet RED96e system (ForteBio, Menlo Park, CA, USA) to assess the affinity between HA and CD44 protein. Briefly, His-CD44 protein (25 μg/mL) was loaded onto a tris-nitriloacetic acid biosensor (ForteBio) and held for 600 s. The biosensor was then placed in binding buffer containing 500 ng/L VNP-HA for 200 s. VNP (500 ng/L) was used as a control.

### 2.10 Cell sorting and culture

The HNSCC cell lines CAL27 and SCC15 were acquired from the American Type Culture Conservation Center (Manassas, VA, USA). CSCs with high aldehyde dehydrogenase activity (ALDH^high^) were sorted from CAL27 and SCC15 cells using the ALDEFLUOR assay kit (STEMCELL Technologies, Vancouver, BC, Canada). ALDH^high^ cells were screened using flow cytometry, and the fluorescence intensity was compared with that of diethylaminobenzaldehyde (DEAB)-treated controls. The ALDH^high^ cells were cultivated in Dulbecco's Modified Eagle Medium (DMEM) supplemented with 1 % antibiotics and 10 % fetal bovine serum (FBS) in a cell culture incubator at 37 °C and 5 % CO_2_. To establish the cisplatin-resistant SCC15-R cell line, SCC15 cells were progressively exposed to increasing concentrations of cisplatin, ultimately maintaining resistance at 3 μM cisplatin. PTC209@VNP-HA was introduced into the cell cultures once the cells achieved 70–80 % confluence.

### 2.11 Cytotoxicity assay

Approximately 1 × 10^4^ cells were plated in each well of a 96-well microplate (Corning, Corning, NY, USA). Following 12 h of incubation, the cells were cultured with various concentrations of nanoparticles ranging from 1 to 200 μg/mL and PTC209 drug concentrations from 0.25 to 10 μmol/L for durations of 24 h. Methyl thiazolyl tetrazolium (MTT) solution was added to each well (10 μL, 5 mg/mL), followed by incubation at 37 °C for 4 h. The MTT was aspirated, and 100 μL of dimethyl sulfoxide was added to solubilize the purple formazan crystals. A microplate reader was used to measure the optical density at a wavelength of 570 nm for cell viability assessment. The control group, representing 100 % cell viability, was maintained in untreated medium. The control and test concentrations were replicated three times to ensure statistical reliability. Cell viability was calculated as follows:

$$Cell\;viability\;\%=\frac{{OD}_{sample}-{OD}_{blank}}{{OD}_{control}-{OD}_{blank}}\times 100\%$$

### 2.12 Cellular delivery assay

To trace nanoparticle distribution, 2.5 mg of rhodamine B isothiocyanate (Macklin Biochemical) was covalently bonded to the remaining amine groups of MSN-HA or VNP-HA after incubation in 25 mL ethanol at 25 °C for 24 h. Covalent bonding was achieved through click reaction of the amine and isothiocyanate groups, which ensured effective labeling for distribution tracking. SCC15 cells (5 × 10^4^ cells per well) were seeded in 24-well plates (NEST Biotechnology, Wuxi, China). Cells were inoculated and walled for 12 h. Cells were incubated with rhodamine-attached MSN-HA (12 μg/mL) and rhodamine-attached VNP-HA (12 μg/mL), respectively, for 4 h. Then, the cells were washed three times with PBS. A 4 % paraformaldehyde fix solution (Biosharp, Hefei, China, 200 μL) was added for 10 min, and then the cells were washed three times with PBS. Then, a solution of 5 % Triton X-100 (SolarBio, Pasig, Philippines) in PBS was added and the cells were incubated for 10 min at room temperature, followed by washing with PBS. A Vari Fluor 488–phalloidin working solution (MedChemExpress) was added (200 μL per well), and the cells were stained for 30 min at room temperature away from direct light. The cells were washed three times with PBS to remove excess phalloidin, and then sliced and sealed with 4’,6-diamidino-2’-phenylindole (DAPI, SolarBio)-containing sealer for confocal laser scanning microscopy (FV3000, Olympus, Tokyo, Japan).

### 2.13 Transwell invasion assay

An invasion assay was performed to assess the invasive ability of CAL27 and SCC15 cells treated with PTC209@VNP-HA by counting invading clones passing through a polycarbonate membrane containing 8-μm pores. The upper chamber of the well plate was coated with matrigel (Corning). Approximately 3 × 10^5^ cells were harvested, suspended in serum-free DMEM, and inoculated into the upper chamber, and an appropriate amount of DMEM was added to the lower chamber. Subsequently, 10 μg/mL PTC209@VNP-HA (containing 2.5 μg/mL PTC209) was added to the upper chamber. The drug content of PTC209 in the free PTC209 and PTC209@MSN-HA experimental groups was consistent with the PTC209@VNP-HA experimental group. Matrigel was scraped off the upper surface of the polycarbonate membrane after 24 h. Cells on the membrane were fixed with 4 % paraformaldehyde solution for 15 min, stained with 0.1 % crystal violet for 15 min, and observed and photographed under a microscope. The cells in nine random areas of the images were counted using ImageJ software (National Institutes of Health, Bethesda, MD, USA).

### 2.14 Comet assays

A single-cell gel electrophoresis (SCGE) comet assay was performed using a kit (ADI-900-166, Enzo Life Sciences, Farmingdale, NY, USA) to detect DNA damage. The cells were embedded in agarose, lysed, and subjected to electrophoresis to separate the DNA into “comet tail” fragments. In this assay, the comet tail length and intensity indicate the damage levels of individual cells, providing insight into environmental and therapeutic effects on DNA integrity. Cells were processed and cell suspensions (1 × 10^5^) were mixed with agarose solution at a volume ratio of 1:50. For each sample, 75 μL of cell suspension was mounted on a pre-warmed slide at 37 °C. The slides were incubated in pre-cooled lysis solution at 4 °C for 40 min, and then transferred to alkaline solution for 40 min at room temperature. To separate the DNA fragments, the slides were electrophoresed (25 V) in Tris/borate/ethylenediaminetetraacetic acid buffer for 15 min. The slides were stained with CYGREEN nucleic acid dye (Enzo Life Sciences) for 30 min, until the DNA became visible, and then were allowed to rest at room temperature for 1 h to allow the agar gel to form a plane and image. Repeat assessments were performed on at least 100 cells per sample using the CASP v1.2.2 analysis tool (СaspLab, Wroclaw, Poland).

### 2.15 Terminal deoxynucleotidyl transferase dUTP nick-end labeling (TUNEL)

A TUNEL kit (T2196, SolarBio) was used to evaluate apoptosis in HNSCC cells. Images were acquired under a fluorescence microscope (Olympus). Positive TUNEL rates were obtained for PTC209@VNP-HA.

### 2.16 Tumor sphere formation assays

In tumor sphere formation assays, ALDH^high^ and ALDH^low^ cells were added to ultra-low-adhesion well plates and cultured in medium containing 1 % N-2 Supplement (Thermo Fisher Scientific), 10 ng/mL human recombinant basic fibroblast growth factor, 20 ng/mL human recombinant epidermal growth factor, and 1 % B-27 Supplement mixed into serum-free DMEM/F12 (Thermo Fisher Scientific). After 12 days of PTC209@VNP-HA treatment, spheres larger than 70 μm in diameter were counted under a microscope. These assays evaluated the effects of PTC209@VNP-HA on cell growth and differentiation.

### 2.17 Western blot analysis

Cells were lysed using radioimmunoprecipitation assay buffer (R0010; SolarBio), and a protease/phosphatase inhibitor cocktail (HX1864; Huaxing Bio, Shanghai, China) was added to prevent protein degradation. The lysed proteins were separated by sodium dodecyl sulfate–polyacrylamide gel electrophoresis (SDS-PAGE) and then transferred to a polyvinylidene difluoride membrane. Non-specific protein binding was blocked with 5 % skim milk for 1 h. Primary antibodies (Cell Signaling Technology, Danvers, MA, USA), including anti-BMI1 (cat. no. 6964, 1:1000), anti-ALDH1 (cat. no. 54135, 1:1000), anti-SOX2 (cat. no. 14962, 1:1000), and anti-GAPDH (cat. no. 2118, 1:1000), were incubated at 4 °C overnight and then labeled with corresponding fluorescence-labeled secondary antibodies. The primary antibody signal was observed using the Clarity Western ECL kit (cat. no. 34577; Thermo Fisher Scientific).

### 2.18 Limiting dilution assay

Female nude mice (6–8 weeks) were obtained from the Department of Animals, Faculty of Medicine, Peking University. All animal experiments were conducted in accordance with the animal experiment protocol approved by the Biomedical Ethics Committee of Peking University. Female nude mice were prepared for dilution transplantation. ALDH^high^ SCC15 CSCs were treated with PTC209@VNP-HA and digested into single-cell suspensions. Equal volumes of matrigel were mixed with varying numbers of treated cells, and the mixtures were injected into the left armpits of nude mice. After 4 weeks, tumors in all mice were photographed and recorded. Data were analyzed using ELDA software (Walter and Eliza Hall Institute of Medical Research, Parkville, Australia).

### 2.19 *In vivo* tumor growth in mice

SCC15 ALDH^high^ CSCs (1 × 10^6^) were inoculated onto the tongues of female BALB/c-nude mice during orthotopic tumor formation. Approximately 1 week later, all mice were randomly divided into seven groups (n = 6), which were treated with 200 μL of control solution, 5 mg/kg PTC209, 60 mg/kg PTC209, 20 mg/kg PTC209@MSN-HA, or 20 mg/kg PTC209@VNP-HA. Injections were administered every 3 days for 3 weeks. Then, the mice were sacrificed and the tongue and cervical lymph nodes were removed. For the cisplatin-resistant human cancer cell line model, SCC15 cisplatin resistance cells (5 × 10^6^) were injected into the left armpits of nude mice. After 1 week of growth, the tumor volume reached approximately 100 mm^3^, and the mice were randomly divided into five groups (n = 6), which were treated with 200 μL of control solution, 3 mg/kg cisplatin, 20 mg/kg PTC209@MSN-HA, 20 mg/kg PTC209@VNP-HA, or 3 mg/kg cisplatin in combination with 20 mg/kg PTC209@VNP-HA. After 3 weeks of treatment, the mice were sacrificed and the tumors were excised and weighed.

### 2.20 4NQO mouse model of HNSCC

Bmi1^CreER^ and Rosa^tdTomato^ mice (C57 mice, aged 6–8 weeks) were provided drinking water containing 4-nitroquinoline N-oxide (4NQO) for 18 weeks to induce the development of HNSCC. Subsequently, they were switched to deionized water for an additional 4 weeks to establish a spontaneous HNSCC model. At 22 weeks, the mice were randomized into five groups (n = 12), which were treated with 200 μL of control solution, 3 mg/kg cisplatin, 20 mg/kg PTC209@VNP-HA, or 3 mg/kg cisplatin in combination with 20 mg/kg PTC209@VNP-HA. Drug administration was conducted every 3 days for a period of 3 weeks. The mice were given tamoxifen to label Bmi1^+^ CSCs prior to sacrifice; following euthanasia, the tongue and cervical lymph nodes (3–4 per mouse) were excised, and lesion surface areas were calculated. For histological analysis, longitudinal sections of the excised tongue and lymph nodes were fixed in 4 % paraformaldehyde overnight. Subsequently, the tissues were dehydrated, embedded in paraffin, and prepared as 5-μm tissue sections.

### 2.21 Immunostaining

Paraffin sections were subjected to antigen repair in citrate buffer (pH = 6). Tissues were incubated overnight at 4 °C with anti-PCK (cat. no. 8018, 1:200; Santa Cruz Biotechnology, Dallas, TX, USA) and anti-BMI1 (cat. no. Sc6964, 1:200; Cell Signaling Technology). For binding to target molecules, horseradish peroxidase-labeled polymer was incubated with the tissues for 60 min at room temperature, using 3,3'-diaminobenzidine (Jinqiao Biotechnology, Zhongshan, China) as a chromogen, which formed a visible precipitate at peroxidase activity sites. Nuclei were stained with hematoxylin for contrast. Antigen repair was performed prior to immunofluorescence, followed by overnight incubation at 4 °C with p-H2A.X antibody (cat. no. 7631, 1:200; Cell Signaling Technology). Next, color development was conducted using Cy2-labeled secondary antibody (Jackson ImmunoResearch Laboratories, West Grove, PA, USA). The nuclei were then stained with DAPI solution. To quantify BMI1^+^ cells, we performed immunohistochemical staining and used ImageJ software to count positively stained cells in five randomly selected fields per slide. Results were expressed as the average number of BMI1^+^ cells per field. The lesion surface area was measured using Image J software by outlining the lesion boundaries on scanned histological images. The area was calculated in square millimeters and averaged across all lesions per animal.

### 2.22 Statistical analysis

GraphPad Prism v9.0 (GraphPad Software, San Diego, CA, USA) was used for all statistical analyses. All *in vitro* experiments were repeated at least twice. Student’s *t*-test was used for comparisons between two groups, and one-way analysis of variance (ANOVA) and the *χ*^2^ test were used for multiple-group comparisons, based on a significance threshold of *P* < 0.05.

## 3 Results

### 3.1 PTC209@VNP-HA synthesis and characterization

**Fig. 1:**
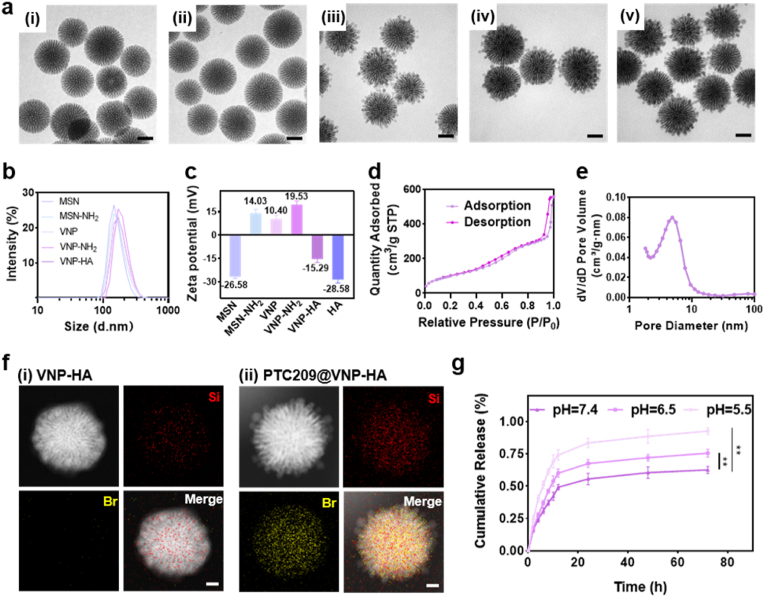
**PTC209@VNP-HA synthesis and characterization.** (a) Transmission electron microscopy (TEM) images showing the nanomorphology of (i) MSN, (ii) MSN-NH_2_, (iii) VNP, (iv) VNP-NH_2_, and (v) VNP-HA. Scale bar: 50 nm. Dynamic light scattering analysis of (b) hydrodynamic diameters in PBS and (c) ζ-potential. (d) Nitrogen sorption isotherm and (e) pore size distribution for VNP-HA. (f) Elemental mapping of (i) VNP-HA and (ii) PTC209@VNP-HA, indicating silicon (Si) and bromine (Br) distribution. Scale bar: 20 nm. (g) Release profiles of PTC209 from PTC209@VNP-HA in PBS buffer with different pH values, ∗∗P < 0.01 using one-way ANOVA.

We developed PTC209@VNP-HA as a drug delivery nanoplatform that contains a PTC209-loaded MSN core with virus-mimicking spines modified by HA. MSNs were synthesized via a modified single-pot hydrothermal protocol [25]. Surface modification of the negatively charged MSNs by amine groups (MSN-NH_2_) using APTES. Shell nanoparticles were prepared using a modified Stöber method allowed electrostatic adsorption onto the MSN-NH_2_ [26]. We obtained VNPs by improving on a method reported in a previous study [27], which assembled a virus-like structure composed of multiple branched nanostructures. Following modification of the VNP amino groups to form VNP-NH_2_, covalent binding of HA onto shell particles formed VNP-HA, which then adsorbed PTC209 onto the mesoporous core to obtain PTC209@VNP-HA. TEM images revealed that both MSN and MSN-NH_2_ retained their uniform morphology and dendritic structure, with no significant change in size (∼110 nm). VNPs were formed by attaching dozens of oriented particles within a narrow size range (∼10 nm; Fig. 1a) to the MSN-NH_2_ surface. The modification of VNPs with APTES (VNP-NH_2_, ∼130 nm) and VNP-NH_2_ with HA (VNP-HA, ∼130 nm) did not alter their virus-mimicking morphology and did not result in significant size changes. The number of shell particles could be adjusted on the MSN core. Varying the feed volume of shell particle solutions from 0.2 to 1.2 mL (Fig. S1) resulted in notable changes in surface morphology. It is noted that insufficient numbers would result in the loss of viral morphology.

The VNP-HA construction was analyzed using dynamic light scattering (Fig. 1b, Table S1); the results showed similar particle size distribution trends to those observed in TEM images. PTC209@VNP-HA and all other nanoparticles exhibited good monodispersibility in PBS. Due to the colloid stability conferred by surface modification with HA [28], VNP-HA exhibited the best dispersibility among tested nanoparticles, with a polydispersity index of 0.15. Changes in ζ-potential guided each step of the nanoparticle synthesis process, with positive ζ-potential measured for MSN-NH_2_ and VNP-NH_2_ (14.03 and 19.53 mV) following MSN (−26.58) and VNP (10.40) surface amino functionalization, and negative ζ-potential measured for VNP-HA (−15.29 mV) after chemical linking with HA (Fig. 1c). Traditional MSNs and solid silica nanoparticles are not biodegradable due to their silica composition, whereas dendritic MSNs possess exceptional biodegradability due to their thin pore walls and amorphous structure [25,29]. The biodegradation of VNP-HA in PBS was assessed in a 28-day experiment. TEM images revealed that VNP-HA degradation progressed from the outer surface to the inner core (Fig. S2). The mass of degraded VNP-HA in PBS reached approximately 56 % by day 28. Achieving optimal biodegradability is essential for extending the treatment duration of VNP-HA and reducing systemic toxicity, both for future research and clinical applications [30].

We performed multiple tests to verify that PTC209 was loaded into PTC209@VNP-HA. TEM images and nitrogen adsorption experiments (Fig. S3) showed that the previous “neck-enhancing” method to form virus-mimicking morphology would reduce the diameter of core MSN mesopores (<3 nm) compared with original MSN (>6 nm). TEM images (Fig. 1a) also showed that after the modified “neck enhancing” method [31], the shell particles were firmly attached to MSN-NH_2_ to form VNP. And to further determine that the virus-mimicking nanoparticles could be drug-carrying, we performed nitrogen adsorption experiments. Nitrogen adsorption experiments conducted to verify that the VNPs could carry the target drug confirmed slight shrinkage of the VNP mesopore (∼5.5 nm) compared to the MSN mesopore, and demonstrated that the mesopore of the final synthesized VNP-HA was further reduced by ∼5 nm compared to the VNP (Fig. 1d, e and S4). SEM images showed that after drug loading, PTC209@VNP-HA was comparable to PTC209 powder mixed with VNP-HA powder, and no drug crystals were visible in the field of view of the PTC209@VNP-HA group, which demonstrated that PTC209 had been fully loaded into the nanoparticles (Fig. S5). Element mapping profiles provided distinct and direct evidence for PTC209 loading. VNP-HA exhibited background signals, whereas PTC209@VNP-HA displayed highly concentrated bromine signals, with no elemental segregation or phase separation, illustrating that bromine atoms were uniformly distributed within the nanostructures (Fig. 1f).

Controlled release of loaded drugs at tumor sites is a critical performance metric for nanoparticles in cancer treatment. We evaluated the release of PTC209 from PTC209@VNP-HA under three pH conditions (7.4, 6.5, and 5.5) (Fig. 1g). Following incubation in a neutral environment (pH = 7.4) for 24 h, PTC209@VNP-HA demonstrated remarkable stability, releasing only ∼20 % of the loaded PTC209 drug. This low level of drug leakage is highly advantageous, as it significantly minimizes the potential for toxic side effects to healthy tissues. In contrast, PTC209 release rates from PTC209@VNP-HA increased dramatically to 64 % and 83 % after 24 h, and then gradually increased to 70 % and 91 % after 72 h, during incubation in acidic environments at pH levels of 6.5 and 5.5, respectively.

### 3.2 Virus-mimicking morphology promotes CSC uptake

**Fig. 2:**
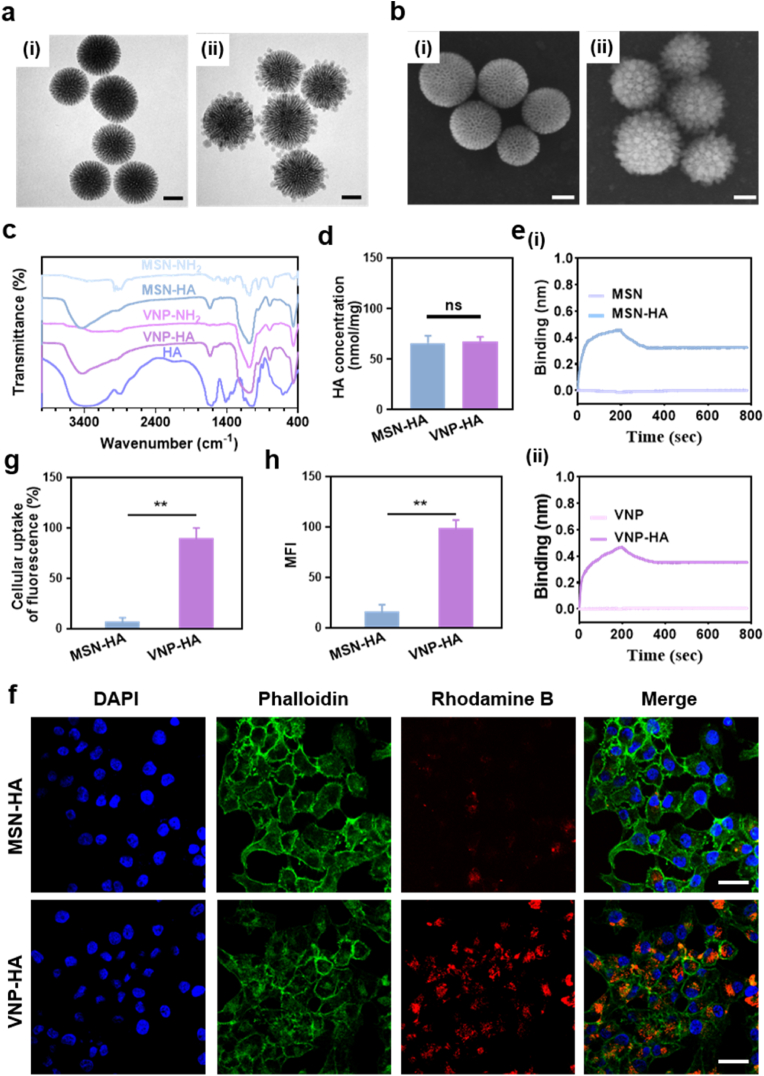
**Virus-mimicking morphology promotes CSC uptake.** (a) TEM images and (b) SEM images showing the nanomorphology of (i) MSN-HA and (ii) VNP-HA. Scale bar: 50 nm. (c) Fourier-transform infrared (FTIR) spectra of MSN-NH_2_, MSN-HA, VNP-NH_2_, VNP-HA, and HA. (d) HA concentrations of MSN-HA and VNP-HA. (e) Biological layer interferometry (BLI) assessment of CD44 protein (human) binding kinetics with (i) MSN-HA and (ii) VNP-HA. (f–h) Confocal microscopic images illustrating MSN-HA and VNP-HA uptake by cells (rhodamine B, red), nuclei (4’,6-diamidino-2’-phenylindole [DAPI], blue), and membranes (Vari Fluor 488–phalloidin, green). Scale bar: 40 μm. Data are proportions of cells taking up nanoparticles. MFI, mean fluorescence intensity. Asterisks indicate significant differences (∗∗*P* < 0.01; unpaired Student’s *t*-test).(For interpretation of the references to colour in this figure legend, the reader is referred to the Web version of this article.)

To determine the effects of nanoparticle morphology on the effectiveness of cargo delivery to CSCs, we fabricated smooth MSN-HA nanoparticles under slightly different reaction conditions (see Supporting Method). TEM (Fig. 2a) and SEM (Fig. 2b) images clearly showed that the MSN-HA and VNP-HA mesopores exhibited distinct outer surface topographies. To confirm successful HA conjugation, we conducted FTIR spectrum analysis. The stretching vibration of the hydroxyl groups was stronger in both MSN-HA and VNP-HA (3522 cm^−1^) than in VNP-NH_2_ (3441 cm^−1^). Stretching vibration from the carboxyl group (-COOH, 1648 cm^−1^) was detected in both HA and the nanoparticles (Fig. 2c). Additionally, TGA showed that the rates of conjugated HA on MSNs and VNPs were ∼3.41 % and ∼3.42 %, respectively (Fig. S6). Using the ninhydrin reaction, we calculated the quantity of HA carried by MSN-HA and VNP-HA; statistical analysis revealed that both had equivalent numbers of HA grafts (Fig. 2d), indicating successful attachment of HA to the nanoparticles.

Previous studies have reported that HA can target CD44 proteins [[32], [33], [34]]; however, whether CD44 proteins can target VNP-HA remained to be investigated. We assessed the binding affinity of VNP-HA to CD44 protein in a BLI assay. To observe interactions between CD44 protein and both MSN-HA and VNP-HA, CD44 protein was immobilized onto a biosensor chip. Compared to nanoparticles without HA, both MSN-HA and VNP-HA exhibited much stronger BLI signals via specific interactions with CD44 protein, indicating that HA-bound nanoparticles were able to target CD44 protein (Fig. 2e and S7).

We conducted fluorescence microscopy to determine the uptake efficiency and distribution of nanoparticles within the cell. To facilitate detection, we incorporated rhodamine B in the nanoparticles as a fluorescent marker for tracking and imaging. SCC15 cells seeded in a 24-well culture plate were incubated with MSN-HA and VNP-HA formulations at specific concentrations for 4 h. VNP-HA exhibited significantly higher cellular uptake than MSN-HA. The fluorescence produced by VNP-HA was approximately 6-fold that of MSN-HA (Fig. 2f–h). Therefore, VNP-HA was expected to be more efficient for anticancer drug delivery, and was selected as the drug vector for subsequent experiments.

### 3.3 PTC209@VNP-HA inhibits HNSCC invasion and promote HNSCC apoptosis *in vitro*

**Fig. 3:**
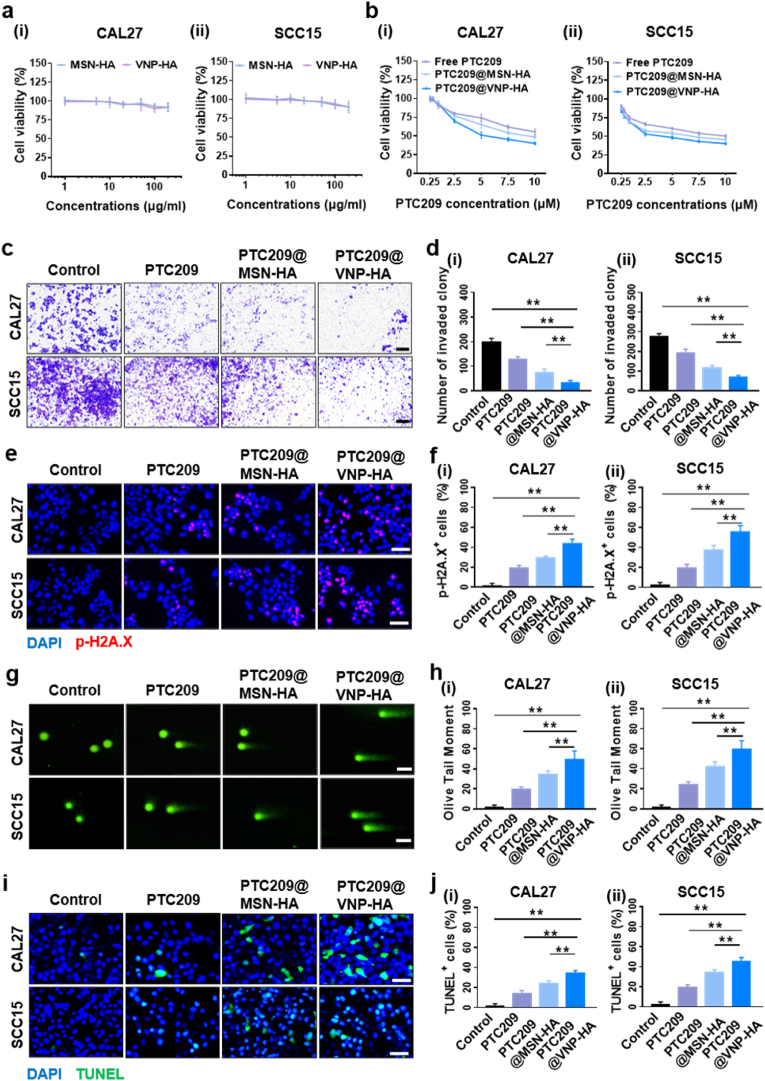
**PTC209@VNP-HA inhibits HNSCC invasion and promote HNSCC apoptosis *in vitro*.** (a) Cytotoxicity of MSN-HA and VNP-HA to (i) CAL27, and (ii) SCC15 cells. The data is normalized to the control. (b) Cytotoxicity of free PTC209, PTC209@MSN-HA, and PTC209@VNP-HA to (i) CAL27 and (ii) SCC15 cells. The data is normalized to the control. (c, d) Cell invasion following treatment to (i) CAL27, and (ii) SCC15 cells with free PTC209, PTC209@MSN-HA, or PTC209@VNP-HA. Scale bar: 200 μm. (e, f) Immunofluorescence imaging and quantitation of p-H2A.X staining (red) to (i) CAL27, and (ii) SCC15 cells. Nuclei were stained using DAPI (blue). Scale bar: 50 μm. (g, h) Images and quantification of DNA comets (>10 cells per group) in (i) CAL27 and (ii) SCC15 cells treated with free PTC209, PTC209@MSN-HA, or PTC209@VNP-HA. Scale bar: 100 μm. (i, j) Apoptosis was determined in (i) CAL27 and (ii) SCC15 cells through terminal deoxynucleotidyl transferase dUTP nick-end labeling (TUNEL) staining at 24 h after treatment. Scale bar: 50 μm. Data are means ± standard deviation (SD). Asterisks indicate significant differences (∗∗*P* < 0.01; one-way ANOVA).

To assess the possible adverse effects of PTC209@VNP-HA, we first evaluated the cytotoxicity of empty nanoparticles *in vitro* at different concentrations. We incubated nanoparticles (MSN, MSN-NH_2_, MSN-HA, VNP, VNP-NH_2_, and VNP-HA) with HNSCC or human umbilical vein endothelial cells (HUVECs) for 24 h. The cell viability results indicated a downward trend as the dose increased, particularly above 100 μg/mL. The survival rate of HNSCC cells treated with VNP-HA dropped below 80 %, whereas that of HUVEC cells increased (Fig. 3a–S8-10). Because normal cells have fewer CD44 receptors on the surface, their uptake of HA-modified nanoparticles was slower, resulting in weaker nanoparticle accumulation and reduced toxicity in normal cells [35]. To investigate whether the encapsulated PTC209 has anti-tumor effects, we treated CAL27 and SCC15 cells with different concentrations (0.25, 0.5, 1, 2.5, 5, 7.5, and 10 μmol PTC209/L) of free PTC209, PTC209@MSN-HA, and PTC209@VNP-HA, respectively. Subsequently, we conducted a MTT cell viability assay and calculated half-maximal inhibitory concentration (IC_50_) values. PTC209@VNP-HA exhibited significantly lower IC_50_ values at 24 h (5 μM) compared to the PTC209@MSN-HA (10 μM), and PTC209 (>10 μM) treatment group (Fig. 3b). These results indicate that PTC209@VNP-HA inhibited the growth of tumor cells. In the following *in vitro* experiments, we selected a PTC209 concentration of 5 μM (below the IC_50_ of PTC209@MSN-HA and free PTC209) for 24 h of cell treatment *in vitro*. Upon intravenous injection, nanoparticles initially interact with erythrocytes. To test the effects of nanoparticles on hemolysis, we incubated mouse erythrocytes with the nanoparticles MSN-NH_2_, MSN-HA, PTC209@MSN-HA, VNP-NH_2_, VNP-HA, and PTC209@VNP-HA, and found that these nanoparticles induced low hemolysis (<5 %). At higher concentrations (100 μg/mL), both MSN-HA and VNP-HA caused a lower hemolysis rate (<4 %) than MSN-NH_2_ or VNP-NH_2_ (Fig. S11). This reduction may be attributed to the HA-modified surfaces, which improve hydrophilicity and reduce the potential harm to erythrocytes [5,36].

Our invasion experiments using PTC209@VNP-HA *in vitro* showed that PTC209@VNP-HA significantly reduced the invasive ability of HNSCC cells relative to free PTC209 and PTC209@MSN-HA (Fig. 3c and d). Immunostaining revealed that cells treated with PTC209@VNP-HA exhibited significantly higher levels of p-H2A.X, a DNA damage marker, than cells treated with free PTC209 or PTC209@MSN-HA (Fig. 3e and f). Next, we conducted a comet assay to detect DNA damage; the results demonstrated that both CAL27 and SCC15 cells treated with PTC209@VNP-HA exhibited significantly longer comet tails than cells treated with free PTC209 or PTC209@MSN-HA (Fig. 3g and h), and that damaged cellular DNA was successfully segregated from intact DNA.

We performed a TUNEL assay to evaluate the relationship between DNA damage and apoptosis, based on the principle that HNSCC cells undergo apoptosis by activating DNA endonucleases that cleave genomic DNA between nucleosomes. The results showed that PTC209@VNP-HA promoted apoptosis via DNA damage in CAL27 and SCC15 cells (Fig. 3i and j).

### 3.4 PTC209@VNP-HA inhibits HNSCC stemness

**Fig. 4:**
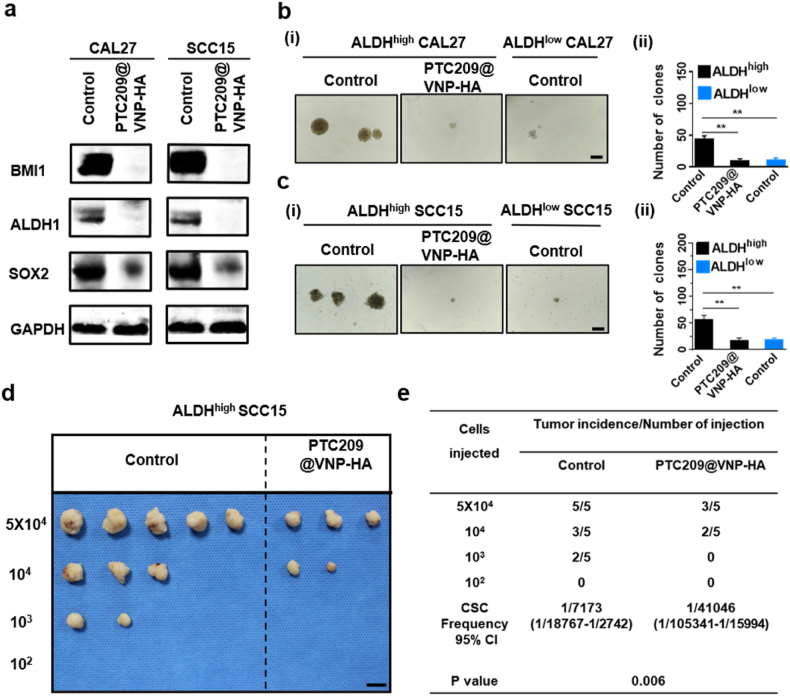
**PTC209@VNP-HA inhibits head and neck squamous cell carcinoma (HNSCC) stemness.** (a) Western blot analysis demonstrated stemness markers after PTC209@VNP-HA treatment. Quantification and visualization of tumor sphere formation by (b) high aldehyde dehydrogenase activity (ALDH^high^) CAL27, and (c) ALDH^high^ SCC15 cells with or without PTC209@VNP-HA treatment. Scale bar: 200 μm. (d, e) Extreme limiting dilution analysis *in vivo* (n = 5). Scale bar: 1 cm. Data are means ± SD. Asterisks indicate significant differences (∗∗*P* < 0.01; Student’s *t*-test).

To demonstrate the effect of VNP-HA on CSCs *in vitro*, we used flow cytometry to isolate ALDH^high^ CSCs from CAL27 and SCC15 cells for subsequent experiments (Fig. S12). We performed several experiments to determine whether PTC209@VNP-HA could eradicate CSCs. First, the levels of various CSC markers were assessed in ALDH^high^ CAL27 and ALDH^high^ SCC15 cells treated with PTC209@VNP-HA. The levels of BMI1, ALDH1, and SOX2 proteins were reduced after treatment (Fig. 4a). Because spheroid formation is an indicator of the self-renewal capacity of CSCs, we performed a tumor ball formation assay to further ascertain whether BMI1 can regulate tumorigenic potential. The results indicated that PTC209@VNP-HA effectively decreased the number of ALDH^high^ cell spheres (Fig. 4b and c). The potent antitumor activity of PTC209@VNP-HA observed *in vitro* inspired us to explore the CSCs inhibitory effects of PTC209@VNP-HA *in vivo*. First, we observed the distribution of nanoparticles *in vivo*. Mice bearing tumors induced by ALDH^high^ SCC15 cells were treated with control solution (PBS), MSN-HA, or VNP-HA via tail vein injection, and an *in vivo* imaging system was used to monitor nanoparticle biodistribution after 1, 12, and 24 h. A fluorescent signal corresponding to VNP-HA was detected at the tumor site after 12 h, and the fluorescent signals were significantly enhanced after 24 h (Fig. S13). Next, we performed *in vivo* dilution experiments to confirm changes in the tumorigenicity of CSCs after PTC209@VNP-HA treatment. The results showed that PTC209@VNP-HA significantly inhibited the tumorigenicity of ALDH^high^ SCC15 cells in nude mice *in vivo* (Fig. 4d and e).

### 3.5 VNP-HA improves the efficacy of PTC209 in orthotopic mice model

**Fig. 5:**
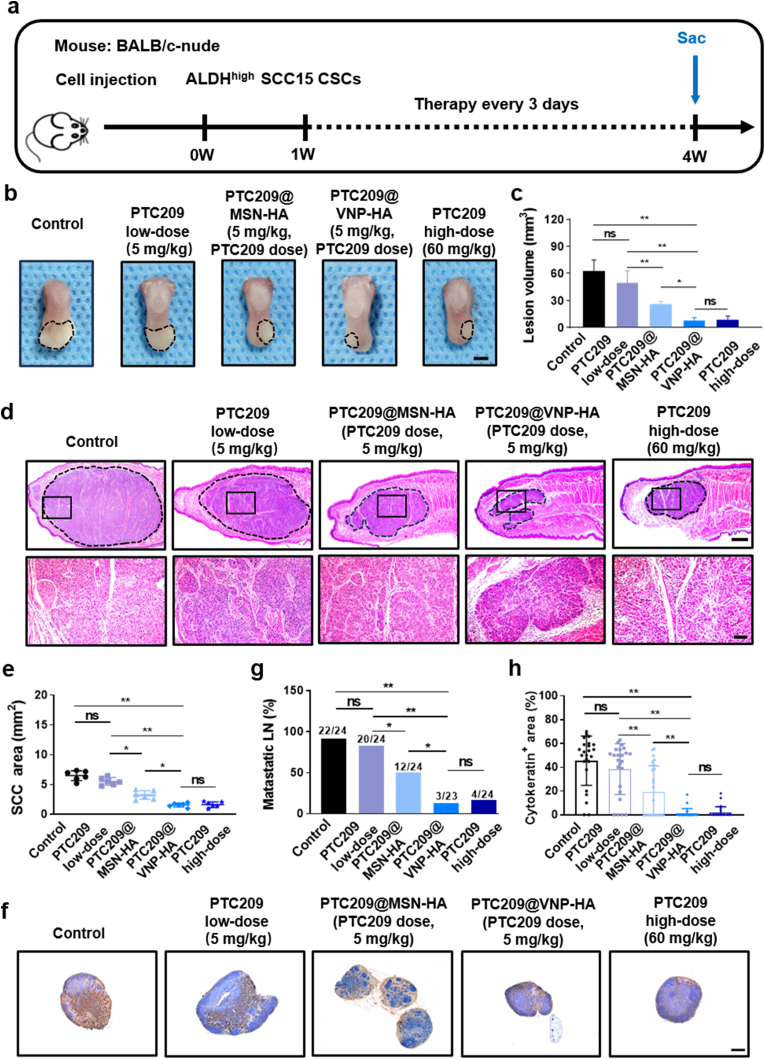
**VNP-HA improves the efficacy of PTC209 in orthotopic mice model.** (a) Diagram of treatment and sacrifice timelines for mice injected with ALDH^high^ CSCs. (b) Tumor images, with lesions circled. Scale bar: 2 mm. (c) Quantification of tumor volume. (d) Hematoxylin and eosin (H & E) staining of HNSCCs. Upper and lower panels show low-magnification (scale bar: 500 μm) and high-magnification (scale bar: 100 μm) images, respectively. (e) Tumor area quantification. (f) Anti-PCK immunostaining of cervical lymph nodes. Scale bar: 200 μm. (g) Metastatic lymph node percentage. (h) Lymph node metastatic area quantification. Data are means ± SD. Asterisks indicate significant differences (∗*P <* 0.05, ∗∗*P* < 0.01). Significance was evaluated using one-way analysis of variance [ANOVA] (c, e, and h) or the χ^2^ test (g).

HNSCC cells, particularly those exhibiting CSC characteristics, frequently migrate and proliferate in cervical lymph nodes. To determine whether PTC209@VNP-HA can impair stemness and metastasis, we employed a mouse orthotopic HNSCC model. ALDH^high^ SCC15 CSCs were inoculated onto the tongues of mice, which were subsequently treated with control solution, PTC209 (low dose, 5 mg/kg), PTC209@MSN-HA (PTC209 dose, 5 mg/kg), or PTC209@VNP-HA (PTC209 dose, 5 mg/kg), PTC209 (high dose, 60 mg/kg) every 3 days for 3 weeks (Fig. 5a). The results showed that PTC209@MSN-HA reduced orthotopic tumor growth, whereas PTC209 low-dose alone did not inhibit tumor growth, similar to the control group. Mice treated with nanomedicine exhibiting virus-like morphology showed a reduction in tumour volume compared to those treated with nanomedicine featuring smooth morphology. PTC209@VNP-HA showed a greater inhibitory effect than low-dose PTC209 (Fig. 5b–e). Cervical lymph nodes were immunostained with anti-PCK to specifically detect lymph node metastasis [37]. The results showed that, compared to low-dose PTC209 alone, both PTC209@MSN-HA and PTC209@VNP-HA significantly reduced lymph node metastasis, and PTC209@VNP-HA reduced ALDH^high^ CSC lymph node metastasis to a greater extent than PTC209@MSN-HA (Fig. 5f–h). In vivo BMI1 immunohistochemical analysis revealed that both PTC209@MSN-HA and PTC209@VNP-HA significantly suppressed BMI1^+^ CSCs compared to low-dose PTC209 alone. Notably, PTC209@VNP-HA exhibited a more pronounced reduction in BMI1^+^ CSCs relative to PTC209@MSN-HA (Fig. S14).

The PTC209 dose used *in vivo* experiments was 5 mg/kg, which was much lower than that administered in our previous study (60 mg/kg) [4]. We compared the antitumor effects of PTC209@VNP-HA and high-dose PTC209 on HNSCCs *in vivo*. The results showed similar effects in the high-dose PTC209 group and the PTC209@VNP-HA group, demonstrating that the proposed VNP design strategy would allow drug dosage reductions, with the potential to increase the potency of PTC209 by 12-fold.

### 3.6 PTC209@VNP-HA overcomes cisplatin resistance in a cisplatin resistance xenograft mouse model

**Fig. 6:**
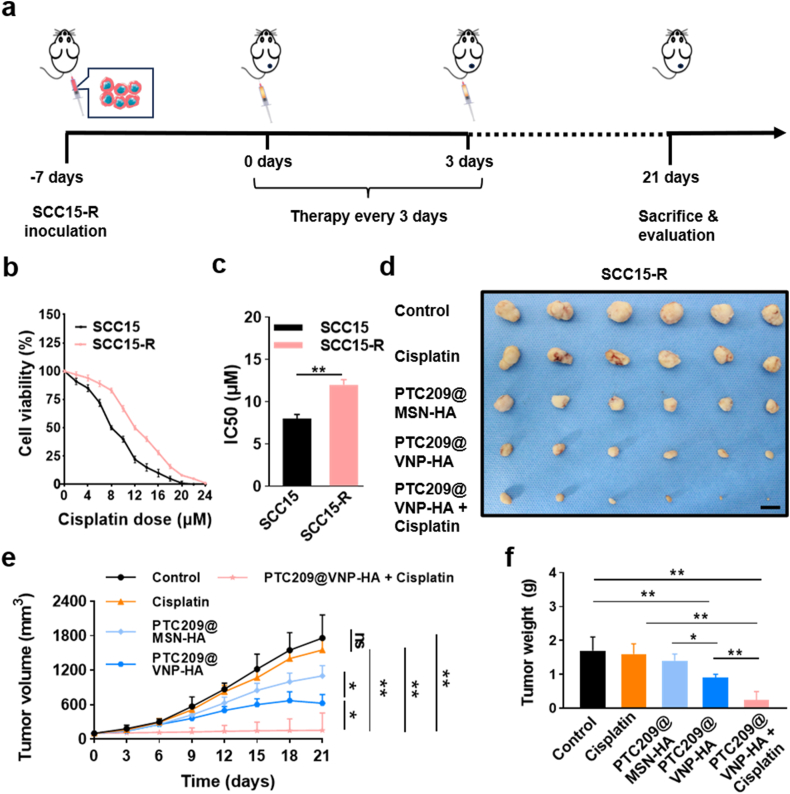
**PTC209@VNP-HA overcomes cisplatin resistance in a cisplatin resistance xenograft mouse model.** (a) Schematic diagram of SCC-R tumor model establishment and treatment. (b)MTT analysis and (c) IC50 of cisplatin treated SCC15 and SCC15 cisplatin-resistant cells. (d) Tumor sample image. Scale bar: 1 cm. (e) Tumor volume growth. (f) Tumor weight. Data are means ± SD. Asterisks indicate significant differences (∗*P* < 0.05, ∗∗*P* < 0.01). Significance was evaluated using unpaired Student’s *t*-test (c) or the one-way analysis of variance [ANOVA] (e, f).

Cisplatin chemotherapy is the primary strategy for treating patients with advanced HNSCC. Despite its widespread use and effectiveness, cisplatin resistance has emerged as a significant challenge. Accumulating evidence has implicated CSCs in resistance to cancer therapy, as well as instances of relapse or recurrence. However, the efficient eradication of CSCs and overcoming chemoresistance remain pivotal challenges. We developed a model for cisplatin resistance following a previously described methodology [38,39]. SCC15 cisplatin resistance (SCC15-R) cells were implanted into the left armpits of nude mice; 1 week later, the mice were treated for 3 weeks with control solution, cisplatin, PTC209@MNS-HA, PTC209@VNP-HA, or PTC209@VNP-HA combined with cisplatin (Fig. 6a). The results indicated that compared with SCC15 cells, SCC15-R cells exhibited enhanced proliferation ability and a higher IC50 value. This confirmed the successful establishment of SCC15-R cell line (Fig. 6b and c). The results indicated that although the cisplatin-resistant model exhibited no responsiveness to cisplatin therapy, virus-mimicking morphology promoted the cancer inhibition efficacy of PTC209 to a greater extent than smooth morphology in the cisplatin-resistant model. Combination of PTC209@VNP-HA plus cisplatin significantly inhibited tumor volume and weight growth compared to the cisplatin alone, suggesting that BMI1 inhibition overcomes cisplatin resistance in HNSCC *in vivo* (Fig. 6d–f). In vivo BMI1 immunohistochemical analysis demonstrated that the population of BMI1^+^ cells was significantly elevated in the cisplatin-treated group compared to the control group. Notably, PTC209@VNP-HA exhibited a more pronounced inhibition of BMI1^+^ cells relative to PTC209@MSN-HA. Furthermore, the combination of PTC209@VNP-HA with cisplatin resulted in a more marked reduction in BMI1^+^ cells compared to either treatment alone (Fig. S15).

### 3.7 Combined PTC209@VNP-HA with cisplatin treatment effectively inhibited HNSCC by eliminating CSCs

**Fig. 7:**
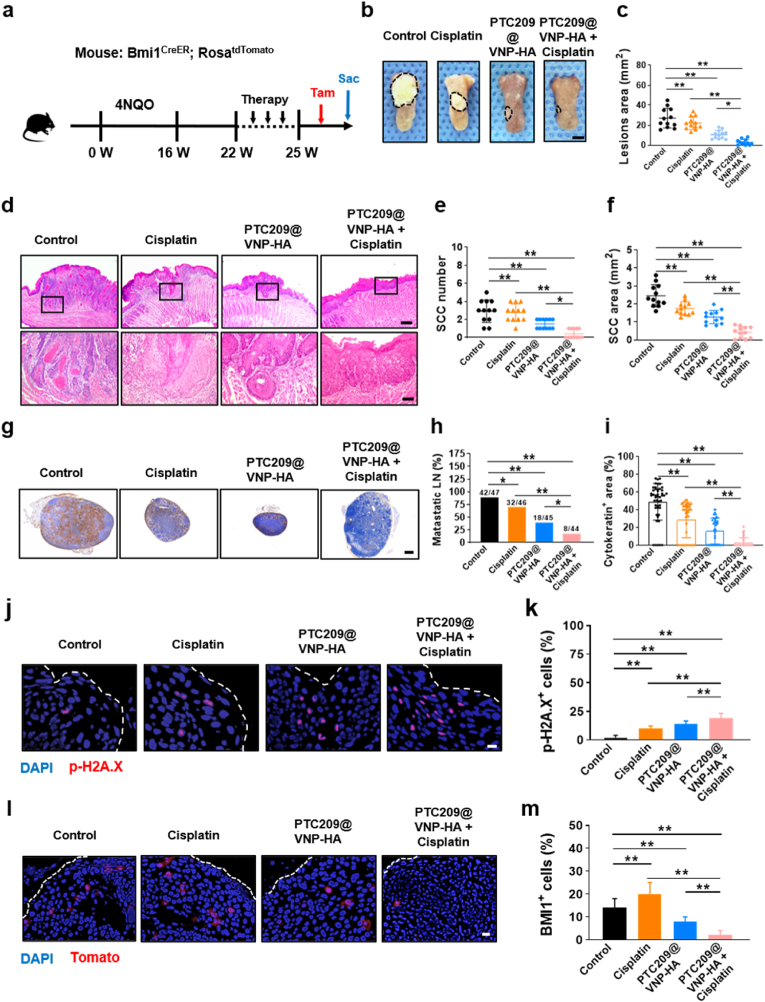
**Combined PTC209@VNP-HA with cisplatin treatment effectively inhibited HNSCC by eliminating CSCs.** (a) Schematic of tamoxifen administration in HNSCC mice to mark Tomato^+^ BMI1^+^ CSCs prior to sacrifice. (b, c) Tongue tumor images; lesions are circled. Scale bar: 2 mm. (d) Representative images of H & E staining of HNSCCs. Upper and lower panels show low-magnification (scale bar: 500 μm) and high-magnification (scale bar: 100 μm) images, respectively. (e, f) Tumor numbers and lesion area quantification (n = 12). (g) Cervical lymph nodes with anti-PCK immunostaining. Scale bar: 200 μm. Quantification of metastatic lymph node (h) percentages and (i) areas. (j, k) Immunofluorescence images and quantification of cells (p-H2A.X, red) and nuclei (DAPI, blue). n = 12. Scale bar: 25 μm. (l, m) Bmi1^+^ Tomato^+^ CSC images and quantification in HNSCC. n = 12. White dotted line indicates the tumor–mesenchymal tissue boundary. Scale bar: 25 μm. Data are means ± SD. Asterisks indicate significant differences (∗*P <* 0.05, ∗∗*P* < 0.01). Significance was evaluated using one-way ANOVA (b, c, e, f, j–m) or the χ^2^ test (h).

Previously, we developed a 4NQO-induced HNSCC mouse model (Rosa^tdTomato^) that was characterized by Bmi1^CreER^, which allowed the undisturbed lineage tracing of CSCs *in vivo* following tamoxifen administration [4]. After consuming the 4NQO inducer for 18 weeks, followed by deionized water for 4 weeks, tumor-bearing mice were treated with control solution, cisplatin, PTC209@VNP-HA, or a combination of PTC209@VNP-HA and cisplatin. On the day preceding euthanasia, mice were administered tamoxifen to mark Tomato^+^ BMI1^+^ CSCs (Fig. 7a). Both Cisplatin group and PTC209@VNP-HA group reduced the tumor lesion area compared to control group. Notably, the combination of PTC209@VNP-HA with cisplatin reduced the lesion area to a significantly greater extent than either cisplatin or PTC209@VNP-HA alone (Fig. 7b and c). Histological assessment showed that PTC209@VNP-HA reduced the number, size, and aggressiveness of HNSCC tumors compared to control. When PTC209@VNP-HA was combined with cisplatin, the number, size, and the SCC area of HNSCC tumors were significantly reduced compared to PTC209@VNP-HA or cisplatin monotherapy (Fig. 7d–f). Next, we examined cervical lymph node metastasis. Anti-PCK immunostaining confirmed that PTC209@VNP-HA reduced lymph node metastasis compared to control. The combination of PTC209@VNP-HA and cisplatin effectively eliminated most lymph node metastatic foci in HNSCC (Fig. 7g–i). Immunostaining revealed that mice treated with the combination treatment exhibited significantly higher levels of p-H2A.X than mice treated with cisplatin and PTC209@VNP-HA alone (Fig. 3j and k). Subsequently, BMI1^+^ CSCs were labeled *in vivo* to assess the efficacy of the combination treatment in eliminating these cells [40]. Consistent with previous studies [39,41], cisplatin was found to enhance the population of BMI1^+^ CSCs in HNSCC. *In vivo* labeling showed that the number of CSCs has increased in HNSCC after cisplatin treatment compared to control. Unlike cisplatin alone, the combination of PTC209@VNP-HA and cisplatin reduced the number of BMI1^+^ CSCs. The combination of PTC209@VNP-HA with cisplatin also significantly improved CSC clearance compared to cisplatin or PTC209@VNP-HA alone (Fig. 7l and m).

**Fig. 8:**
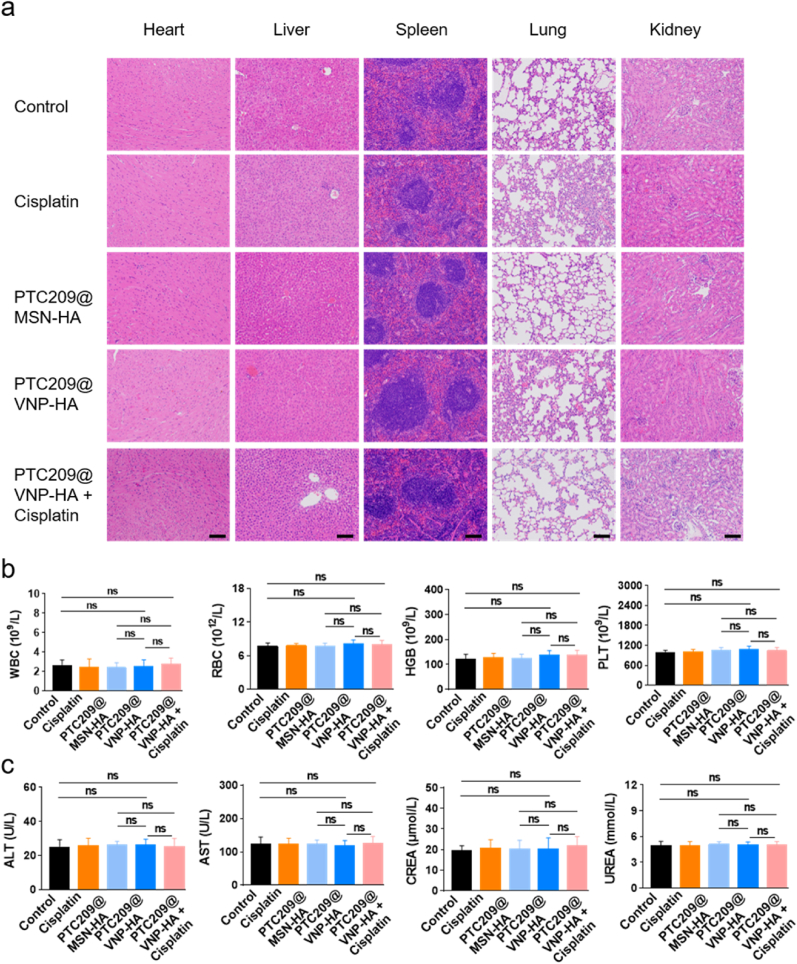
**In vivo biosafety studies.** (a) Histological analysis of nude mice organs from five groups, control, Cisplatin, PTC209@MSN-HA, PTC209@VNP-HA, and PTC209@VNP-HA plus Cisplatin. H&E staining of heart, liver, spleen, lung and kidney, indicating all the therapeutics have good biocompatibility. Scale bar: 100 μm. (b, c) The changes of (b) serum routine blood and (c) blood biochemical of serum samples on Nude mice after different treatment. Data represent the mean ± SD from three mice. No significant difference between the control group and treatment group was noted by one-way ANOVA.

Next, we assessed the safety of PTC209@VNP-HA in nude mice. Histopathological examinations revealed no tissue damage in the heart, liver, spleen, lungs, or kidneys following the administration of PTC209@VNP-HA. Assessments of standard hematological and blood chemistry parameters indicated that the mice exhibited good tolerance to PTC209@VNP-HA (Fig. 8).

## 4 Discussion

The CSC tumor cell subpopulation exhibits self-renewal and differentiation potential, and plays a crucial role in several key aspects of tumor growth, invasion, metastasis, and resistance to chemotherapy [42]. CSCs are capable of driving tumor formation and sustained expansion, and contribute to malignant tumor progression through complex molecular mechanisms. PTC209 is a well-documented small-molecule inhibitor that can abolish self-renewal in CSCs [43]. Although PTC209 significantly inhibits tumor progression alone or in combination with other treatments, challenges associated with immunogenicity, off-target gene effects, and dose-limiting toxicity have hindered its wide application *in vivo* [44].

The role of virus-mimicking morphology in drug delivery has garnered significant attention due to its unique advantages in enhancing cellular uptake, immune evasion, and targeted delivery. Recent studies on virus-like nanoparticles (VLPs) have further underscored the importance of structural mimicry in optimizing therapeutic outcomes. For instance, hepatitis B virus (HBV)-inspired VLPs have demonstrated enhanced liver-specific targeting and efficient gene delivery, attributed to their natural tropism and surface protein interactions [45]. Similarly, influenza virus-mimicking nanoparticles, engineered with hemagglutinin glycoproteins, have shown improved endosomal escape and cytosolic delivery, a critical step for successful intracellular drug release [46]. Additionally, bacteriophage-inspired nanoparticles, such as MS2 VLPs, have been utilized for targeted cancer therapy, leveraging their ability to bind and internalize into tumor cells via receptor-mediated pathways [47]. These findings collectively highlight the versatility of virus-like architectures in overcoming biological barriers and improving therapeutic efficacy.

In this study, we designed VNP-HA for the delivery of PTC209 as a high-efficacy CSC-targeted HNSCC therapy. Although viral vectors have been employed to treat cancer, there remain concerns relating to immunogenicity and mutagenesis risks [22]. Consequently, the development of non-viral vectors with virus-like morphology may create promising tools for achieving an appropriate balance of safety and effectiveness. VNP-HA offers several advantages in enhancing the therapeutic effect of PTC209, including its biocompatibility. Silicon dioxide nanoparticles are widely used in nanocarrier design, and both *in vivo* and *in vitro* experiments have demonstrated the almost negligible cytotoxicity of VNP-HA. Hemolysis testing showed that VNP-HA has almost no toxicity to blood cells in circulation *in vivo*. To ensure that VNP-HA can easily reach tumor tissue via blood circulation, factors such as the size and surface charge of the nanoparticles are also important. The size of VNP-HA is around 130 nm, which appears to be ideal for leveraging the EPR effect for accumulation and selective retention in HNSCC [48]. VNP-HA is negatively charged and binds relatively weakly to blood proteins, which contributes to its long-term circulation in the blood [49]. In addition, virus-mimicking morphology enhances the cellular uptake efficacy of nanoparticles, improving cellular delivery performance [23]. Our orthotopic tumor model demonstrated that the virus-mimicking morphology of PTC209@VNP-HA promoted cellular uptake and enhanced the efficacy of PTC209 compared to PTC209@MSN-HA. Finally, VNP surfaces can easily become functionalized through HA modification to enhance CSC targeting precision [50,51]. Our analyses showed that HA was successfully grafted onto the nanoparticles. Molecular affinity assays demonstrated that VNP-HA had successfully targeted the CD44 receptor protein.

A previous study indicated that cisplatin therapy primarily eliminates proliferating cells, leaving CSCs unaffected [52]. We demonstrated that PTC209@VNP-HA effectively inhibited tumor growth and metastasis by eliminating CSCs through the DNA damage/apoptosis pathway both *in vitro* and *in vivo*. Although PTC209@VNP-HA inhibited growth and metastasis in HNSCC, a prior study demonstrated that specific cytokines or growth factors can lead to the reprogramming or redifferentiation of non-stem tumor cells into CSCs [53]. Combined treatment with PTC209@VNP-HA and cisplatin effectively eliminated both CSCs and non-CSCs, achieving optimal therapeutic outcomes.

In summary, VNP-HA possesses multifaceted functionality, high stability, minimal toxicity, favorable biodegradability, and biocompatibility. Our *in vitro* and *in vivo* experiments demonstrated that PTC209@VNP-HA can effectively eliminate CSCs, prevent metastasis, and overcome cisplatin resistance in HNSCC, highlighting its significant research value and clinical translation potential. However, further investigation of PTC209@VNP-HA is necessary. The stroma of HNSCC includes a complex mixture of infiltrating inflammatory cells such as lymphocytes and macrophages, which play pivotal roles in the tumor microenvironment by releasing a variety of cytokines and growth factors, which in turn have the potential to activate oncogenic signaling pathways, potentially reprogramming or dedifferentiating tumor cells into CSCs. The effects of PTC209@VNP-HA on the cancer immune system also deserve further exploration. We are confident that the proposed virus-mimicking strategy will offer valuable inspiration for designing innovative bionanomaterial drug delivery platforms and present a promising therapeutic approach for sustainable CSC elimination and tumor regression.

## 5 Conclusion

We developed a targeted biomimetic nanomedicine that simultaneously addresses the three major problems of tumor stemness, side effects related to PTC209, and cisplatin resistance. VNP-HA improves cellular uptake efficiency by mimicking the physical surface properties of biological systems, and can directly target CSCs. PTC209@VNP-HA protects healthy tissues while effectively amplifying the response against CSCs, inhibiting tumor proliferation and metastasis, and mitigating cisplatin resistance. This study provides insights into the synthesis of efficient drug-carrying platforms and offers a new biomimetic strategy for eliminating CSCs.

## CRediT authorship contribution statement

**Yiwen Chen:** Writing – review & editing, Writing – original draft, Visualization, Validation, Software, Methodology, Investigation, Formal analysis, Data curation. **Zhen Qin:** Writing – review & editing, Writing – original draft, Methodology, Investigation, Data curation. **Yujia Wang:** Writing – review & editing, Writing – original draft, Methodology, Investigation. **Baoxin Gu:** Methodology, Investigation, Formal analysis. **Jing Wang:** Methodology, Investigation, Formal analysis. **Yunfei Zheng:** Writing – review & editing, Writing – original draft, Conceptualization. **Yuting Niu:** Writing – review & editing, Writing – original draft, Funding acquisition, Conceptualization. **Lingfei Jia:** Writing – review & editing, Writing – original draft, Funding acquisition, Conceptualization.

## Declaration of competing interest

The authors declare that they have no known competing financial interests or personal relationships that could have appeared to influence the work reported in this paper.

## Data Availability

Data will be made available on request.
