# Supplementary material for: CD44-targeted virus-mimicking nanomedicine eliminates cancer stem cells and mitigates chemoresistance in head and neck squamous cell carcinoma
Source: Mater Today Bio. 2025 Mar 29;32:101721. doi: 10.1016/j.mtbio.2025.101721 (PMC12002834; doi:10.1016/j.mtbio.2025.101721)
Supplement: Multimedia component 1 [file mmc1.docx]

**Supporting Information**

**CD44-targeted virus-mimicking nanomedicine eliminates cancer stem cells and mitigates chemoresistance in head and neck squamous cell carcinoma**

*Yiwen Chen^a,b^, Zhen Qin^a^, Yujia Wang^a^, Baoxin Gu^a^, Jing Wang^c^, Yunfei Zheng^d^*, Yuting Niu^e^*, Lingfei Jia**^a, b, f^**

^a^Department of Oral and Maxillofacial Surgery, Peking University School and Hospital of Stomatology, Beijing 100081, PR China.

^b^National Center for Stomatology & National Clinical Research Center for Oral Diseases & National Engineering Research Center of Oral Biomaterials and Digital Medical Devices, Beijing 100081, PR China.

^c^State Key Laboratory of Natural and Biomimetic Drugs, Peking University，Beijing 100091, PR China

^d^Department of Orthodontics, Peking University School and Hospital of Stomatology, Beijing 100081, PR China

^e^Central Laboratory, Peking University School and Hospital of Stomatology, Beijing 100081, PR China

^f^Beijing Advanced Center of Cellular Homeostasis and Aging-Related Diseases，Institute of Advanced Clinical Medicine, Peking University, Beijing 100091, PR China.

***Corresponding authors:**

Prof. Lingfei Jia, [jialingfei1984@sina.com](mailto:jialingfei1984@sina.com)

Prof. Yuting Niu, [yutin.niu@bjmu.edu.cn](mailto:yutin.niu@bjmu.edu.cn)

Prof. Yunfei Zheng, [yunfei_zheng@bjmu.edu.cn](mailto:yunfei_zheng@bjmu.edu.cn)

**Supporting methods**

**Synthesis of MSN-HA**

The carboxyl groups of HA (700 Da, 32 mg) were activated using 1-(3-dimethylaminopropyl)-3-ethylcarbodiimide hydrochloride (EDC, 12 mg) as a catalyst in phosphate-buffered saline (PBS, pH 7.4, 25 mL) at room temperature for a duration of 0.5 h. N-hydroxysuccinimide (NHS, 14 mg) was added and the mixture was stirred continuously for another 0.5 h. Then, MSN-NH_2_ (100 mg) in PBS (pH = 7.4, 25 mL) was added, and the NHS-activated HA was further reacted for 3 h. The MSN-HA product was washed three times with deionized water (20 mL per wash) and ethanol (20 mL per wash) to remove unreacted chemicals. The final targeting delivery system, MSN-HA, was maintained under wet conditions at 4°C.

**Amine group and attached-HA quantification**

Briefly, 100 μg of MSN, MSN-NH_2_, VNP, VNP-NH_2_ and VNP-HA were first dispersed in 100 μL of DI water, respectively. Then, 100 μL of (2 w/v%) DMSO was mixed for chromogenic reaction at 90 ℃ for 20 min. After the mixtures were cooled down to room temperature, 800 μL of ethanol was added to stop the reaction, and the absorbance at 570 nm using a microplate reader (ELX808, BioTek) was recorded. Glycine solutions from 2.5 to 25 mM were used to draw a standard curve. After grafting HA on amino-modified nanoparticles, we calculated the remaining amino content by ninhydrin reaction. The amount of grafted HA was calculated by comparing the remaining amino content with the amino content before the HA was grafted.

**Calculation of molecular weight of nanoparticles**

We divide the volume of nanoparticles into two parts: the pore volume (Vp) and the solid silica volume (Vs) whose density ρ = 2.2 g/cm^3^. Vp value was given by BET data for MSN (0.9 cm^3^/g, ~110 nm), VNP (0.58 cm^3^/g , ~130 nm). For 1g of MSN, VNP,

$$\rho MSN = 1g/(Vp\times1g+Vs) = 1g/(0.9\times1g+1g/2.2) = 0.738 g/\mathrm{cm}^{3}$$

$$\rho VNP = 1g/(Vp\times1g+Vs) = 1g/(0.58\times1g+1g/2.2) = 0.967 g/\mathrm{cm}^{3}$$

Volume of ball = $\frac{4}{3}\times\pi{\times r}^{3}$, Avogadro's constant (N_A_) = 6.02×10^23^.

The molecular weight of MSN nanosphere (M_MSN_) =

ρ_MSN_×V_MSN_× N_A_ = 0.738×$\frac{4}{3}\times3.14{\times\left( 55 \right)}^{3}$×6.02×10^23^ ≈ 309463 g/mol

The molecular weight of VNP nanosphere (M_VNP_) =

ρ_VNP_×V_VNP_× N_A_ = 0.967×$\frac{4}{3}\times3.14{\times\left( 65 \right)}^{3}$×6.02×10^23^ ≈ 669316 g/mol

For 1mg of MSN, the number of nanospheres n ≈ 3.23 nmol. For 1mg of VNP, the number of nanospheres n ≈ 1.49 nmol. For the following calculation, the number of other nanoparticles was converted from MSN or VNP, based on the TGA data.

**Degradation of MSN-HA and VNP-HA**

To evaluate the degradability, the specimens of nanoparticles were dispersed in 10 mL of PBS at a concentration of 0.5 mg/mL, under constant stirring at 37°C for 28 days. On day 1, 7, 14 and 28, nanoparticle samples were collected for TEM observations. Moreover, 5 mL of the specimens were picked up and centrifuged, and the supernatant was subjected to inductively coupled plasma optical emission spectrometry (ICPOES) to determine the concentration of silica in PBS, which accumulates with the process of material degradation. The sediments were put back after redispersion in 5 mL of fresh PBS, continuing the degradation process.

**Hemolysis assay**

Hemolysis tests were carried out to evaluate the toxicity of MSN-NH_2_, MSN-HA, VNP-NH_2_, VNP-HA, PTC209@MSN-HA, and PTC209@VNP-HA to RBCs, respectively. The whole blood from 6-to-8-week-old female ICR mice was obtained via cardiac puncture using syringes and preconditioned in an EDTA anticoagulant tube. Erythrocytes were extracted by centrifugation at 2000 g for 5 minutes at 4 °C and washed three times with PBS to remove serum proteins and the buffy coat. Then, 100 μL of the washed erythrocytes were added to 900 μl of VNP-NH_2_ and VNP-HA suspensions in PBS with final concentrations varied from 1 to 200 μg/mL, individually. The whole system was gently mixed and kept at 37 °C for 1 hour with intermittent shaking, followed by centrifugation (2000 g, 5 min) and spectrophotometric analysis of supernatant at 540 nm wavelength. The released hemoglobin in the supernatant is proportional to the optical density. Erythrocytes incubated in PBS and DI water were used as negative and positive controls, respectively. The hemolysis was evaluated via the following equation:

$Hemolysis \%=\frac{A_{sample} - A_{negative control}}{A_{positive control} - A_{negative control}}$ × 100%

**Ex vivo fluorescence imaging**

To better understand the pharmacokinetics of different therapeutics, their biodistribution was monitored. ALDH^high^ SCC15 cells were injected subcutaneously into the left axilla of nude mice to prepare xenograft mouse model. Briefly, 400 μg of MSN-HA and VNP-HA prepared by MSN separately were added to 200 μL of physiological saline, respectively. Nanoparticles were injected through the tail vein of the subcutaneous tumor model in female nude mice, and 200 μL of saline was injected as a control group. At each time point (1 h, 12 h, 24 h after injection), three mice in each group were euthanized, and their internal organs (heart, liver, spleen, lung, and kidneys) and tumor were collected. The samples were subjected to ex vivo imaging and quantitative analysis by an IVIS Spectrum In Vivo Imaging System (PerkinElmer).

**In vivo biosafety studies.**

To further evaluate in vivo toxicity, the excised organ samples (heart, liver, spleen, lung and kidneys) of each mouse (BALB/c-nude) were fixed in 10% formaldehyde and subjected to hematoxylin and eosin (H&E) staining. The specimens were observed using a light microscope (Olympus). Blood was obtained via the medial canthus vein (the eye socket) of mice after the different treatments. Routine blood and Blood biochemical indicators tests were then conducted in a clinical laboratory to evaluate blood system, liver and kidney functions.

**Supporting tables**

**Table S1.** Hydrodynamic diameters, PDI and ZP of MSN, MSN-NH_2_, VNP, VNP-NH_2_ and VNP-HA. HD: hydrodynamic diameter; PDI: polydispersity index.

|  | **TEM**  **Size (nm)** | **In PBS**  **HD (nm)** | **PDI** |
| --- | --- | --- | --- |
| **MSN** | 110 ± 10.12 | 150.6 ± 0.69 | 0.21 |
| **MSN-NH_2_** | 110 ± 10.59 | 153.4 ± 2.23 | 0.42 |
| **MSN-HA** | 110 ± 10.59 | 154 ± 3.03 | 0.15 |
| **VNP** | 130 ± 10.12 | 168.1 ± 3.38 | 0.34 |
| **VNP-NH_2_** | 130 ± 10.13 | 184.2 ± 4.14 | 0.41 |
| **VNP-HA** | 130 ± 10.58 | 156.8 ± 4.14 | 0.15 |

**Table S2.** Approximate IC50 (μM) of PTC209 formulations in CAL27 cells and SCC15 cells

| **Groups**  **Cells** | **Free PTC209** | **PTC209@**  **MSN-HA** | **PTC209@**  **VNP-HA** |
| --- | --- | --- | --- |
| **CAL27** | >10 | 10 | 5 |
| **SCC15** | >10 | 10 | 5 |

**Supporting figures**


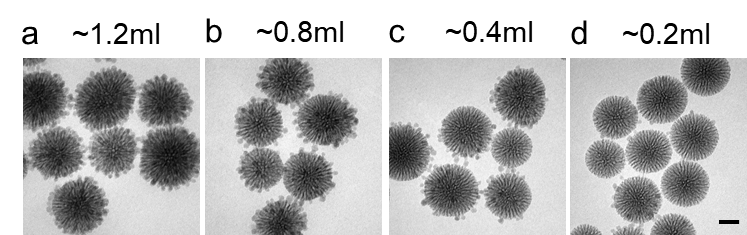


**Fig. S1.** Morphology adjustment of virus-mimicking nanoparticles. After fabricating the virus-mimicking nanoparticles by mixing shell particles with core particles, morphology changes were traced by TEM images. On adjusting the feed volumes of shell particle solutions from (a) 1.2 ml to (b) 0.8 ml, to (c) 0.4 ml and finally to (d) 0.2 ml, the surface morphologies of MSN-NH_2_ (5 mg) changed significantly, with the best sample being obtained when the feed volume was 0.8 ml. Scale bar: 50 nm.


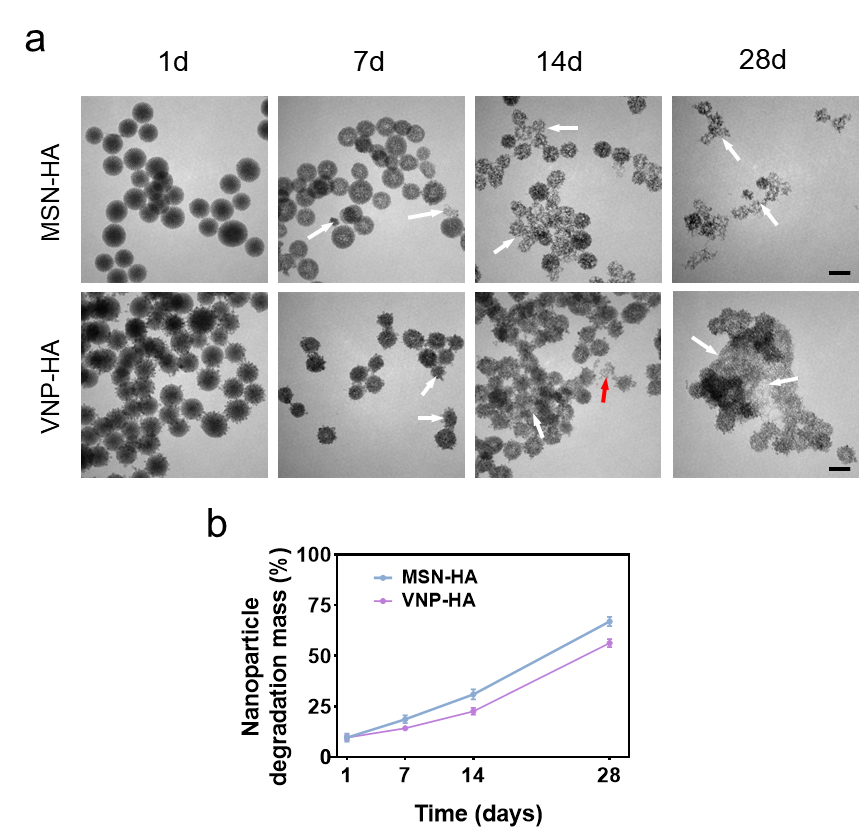


**Fig. S2.** (a) Degradability of VNP-HA in PBS. The white arrows indicate some typical degrading nanoparticles. Red arrows indicate shell particles that have fallen off the virus-like nanoparticles. Scale bar: 100 nm. (b) Quantitative degradation curves of VNP-HA in PBS at different incubation intervals by silica mass.


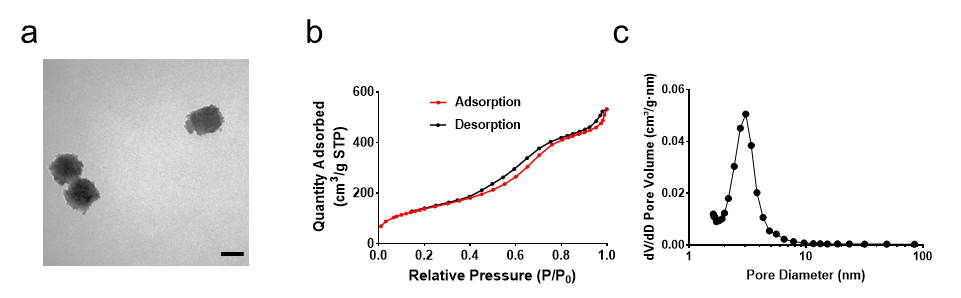


**Fig. S3.** (a) TEM images of VNP prepared with the “neck enhancing” method in previous studies [1]. Scale bar: 100 nm. (b, c) Characterization of (b) nitrogen sorption isotherm and (c) pore size distribution.


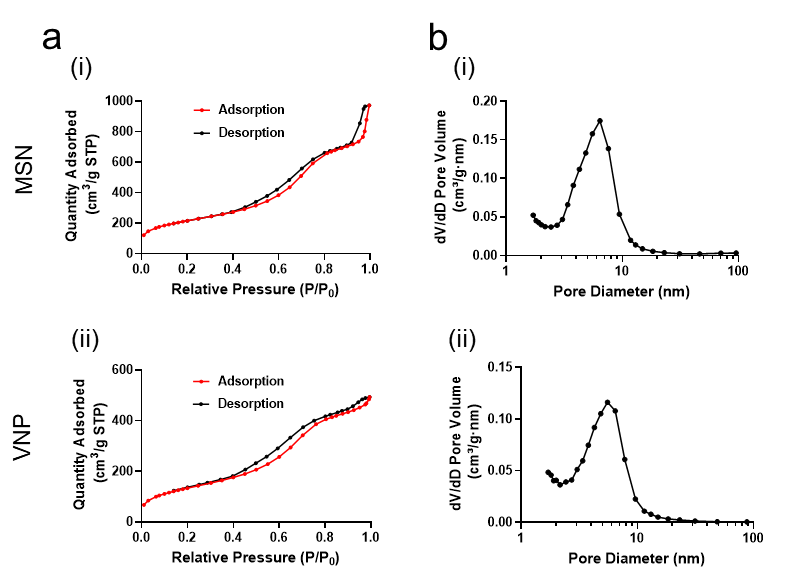


**Fig. S4.** Characterization of (a) nitrogen sorption isotherm and (b) pore size distribution of (i) MSN and (ii) VNP, respectively.


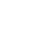


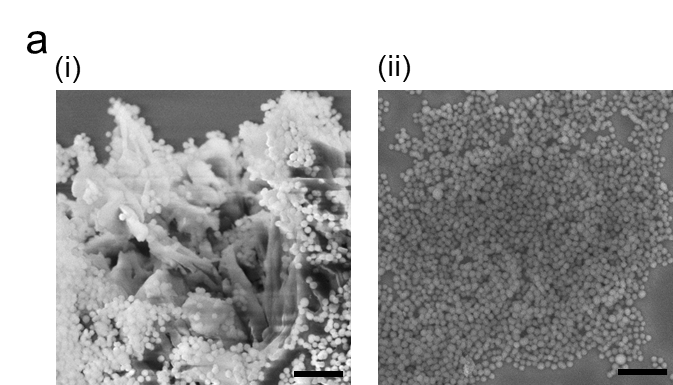


**Fig. S5.** (a) SEM images of (i) VNP-HA powder mixed with PTC209 powder and (ii) PTC209@VNP-HA. Scale bar: 1 μm.


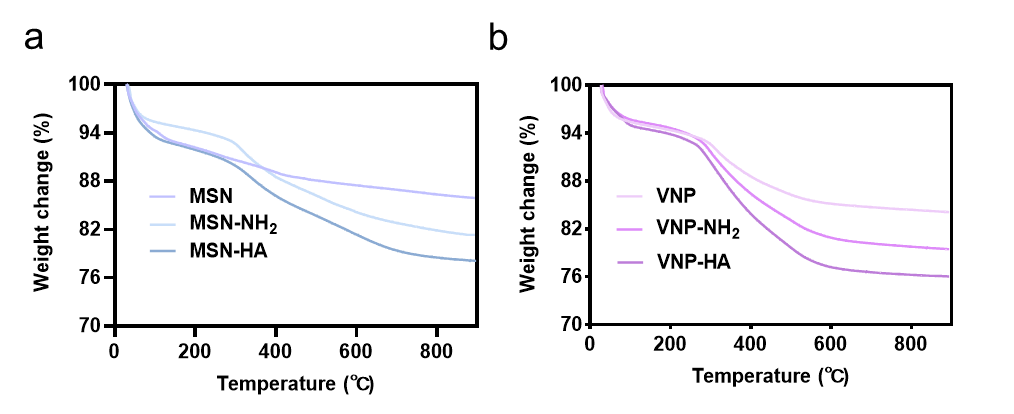


**Fig. S6.** (a) TGA profiles of MSN, MSN-NH_2_ and MSN-HA. (b) TGA profiles of VNP, VNP-NH_2_ and VNP-HA. The quantities of MSN conjugated amino groups and HA were found to be approximately 4.59% and 3.32%, respectively. The quantities of VNP conjugated amino groups and HA were found to be approximately 4.64% and 3.42%, respectively.


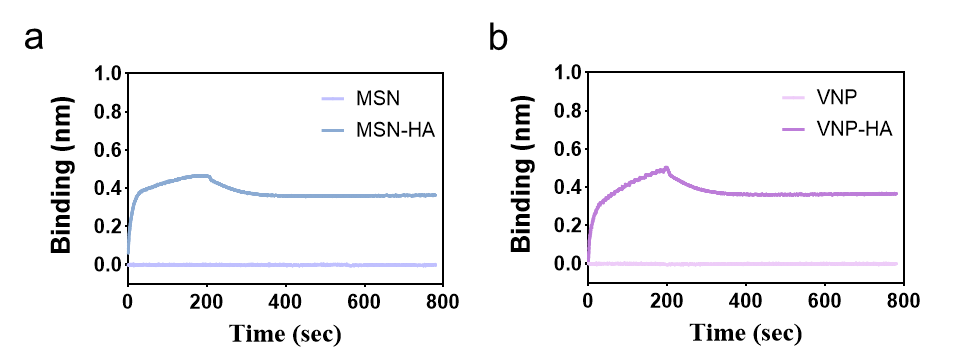


**Fig. S7.** BLI evaluation of the binding performance of CD44 protein (mouse) with (a) MSN-HA and (b) VNP-HA, where CD44 protein was fixed on biosensors.


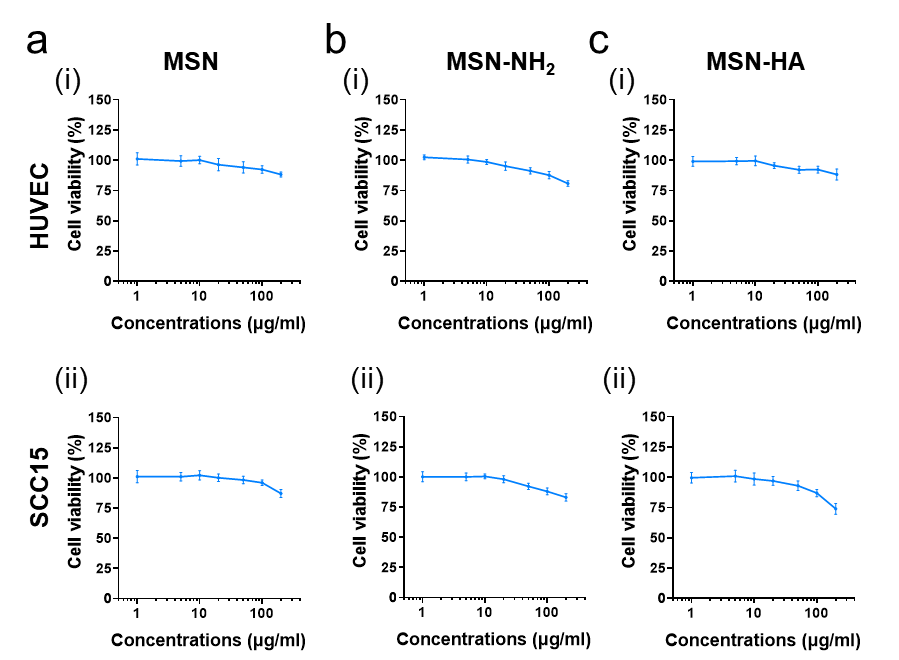


**Fig. S8.** Cytotoxicity of (a) MSN, (b) MSN-NH_2_, and (c) MSN-HA in (i) SCC15 and (ii) HUVEC cells.


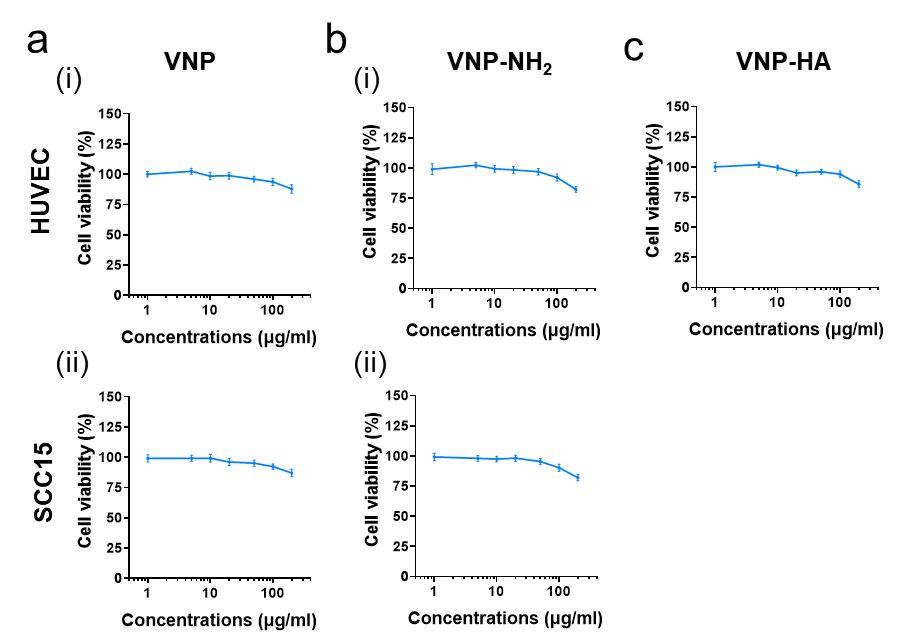


**Fig. S9.** Cytotoxicity of (a) VNP and (b) VNP-NH_2_ in (i) SCC15 and (ii) HUVEC cells. (c) Cytotoxicity of VNP-HA in HUVEC cells.


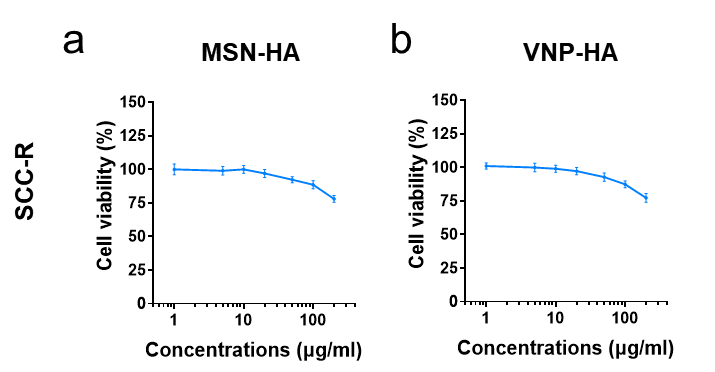


**Fig. S10.** Cytotoxicity of (a) MSN-HA and (b) VNP-HA in SCC15 cells with cisplatin resistance.


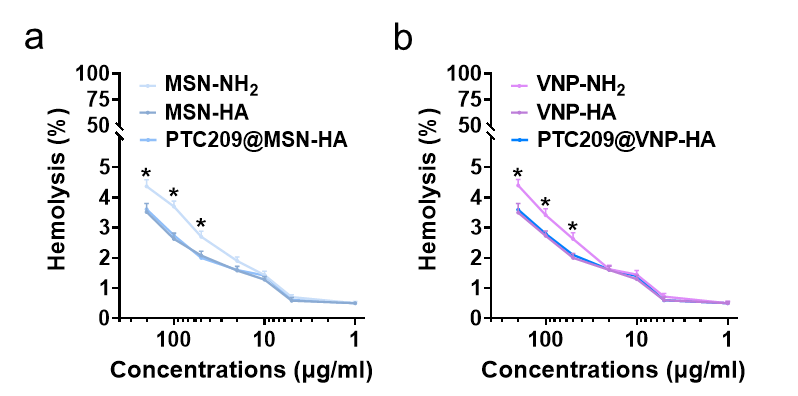


**Fig. S11.** Hemolysis of (a) MSN-NH_2_, MSN-HA, PTC209@MSN-HA, (b) VNP-NH_2_, VNP-HA, and PTC209@VNP-HA on erythrocytes in ICR mice. *P < 0.05 using one-way ANOVA.


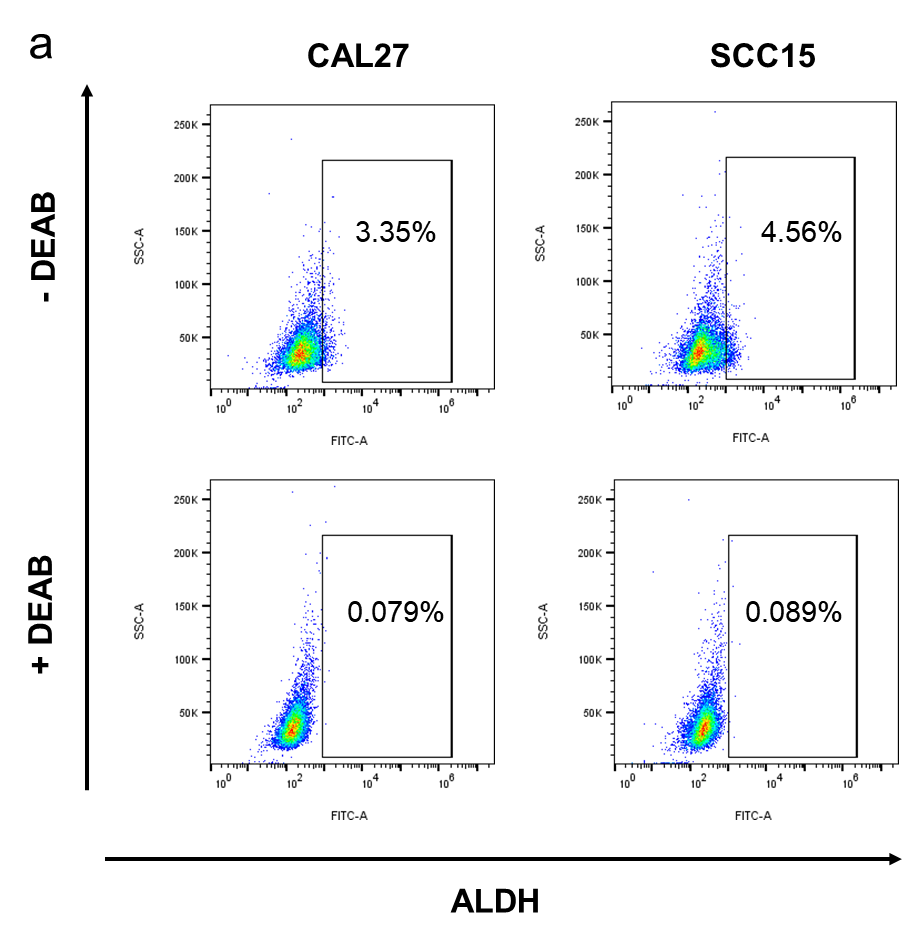


**Fig. S12.** (a) ALDH^high^ cells obtained from CAL27 and SCC15 cells sorting by FACS. A specific inhibitor of ALDH, diethylaminobenzaldehyde (DEAB), was used to control for background fluorescence.


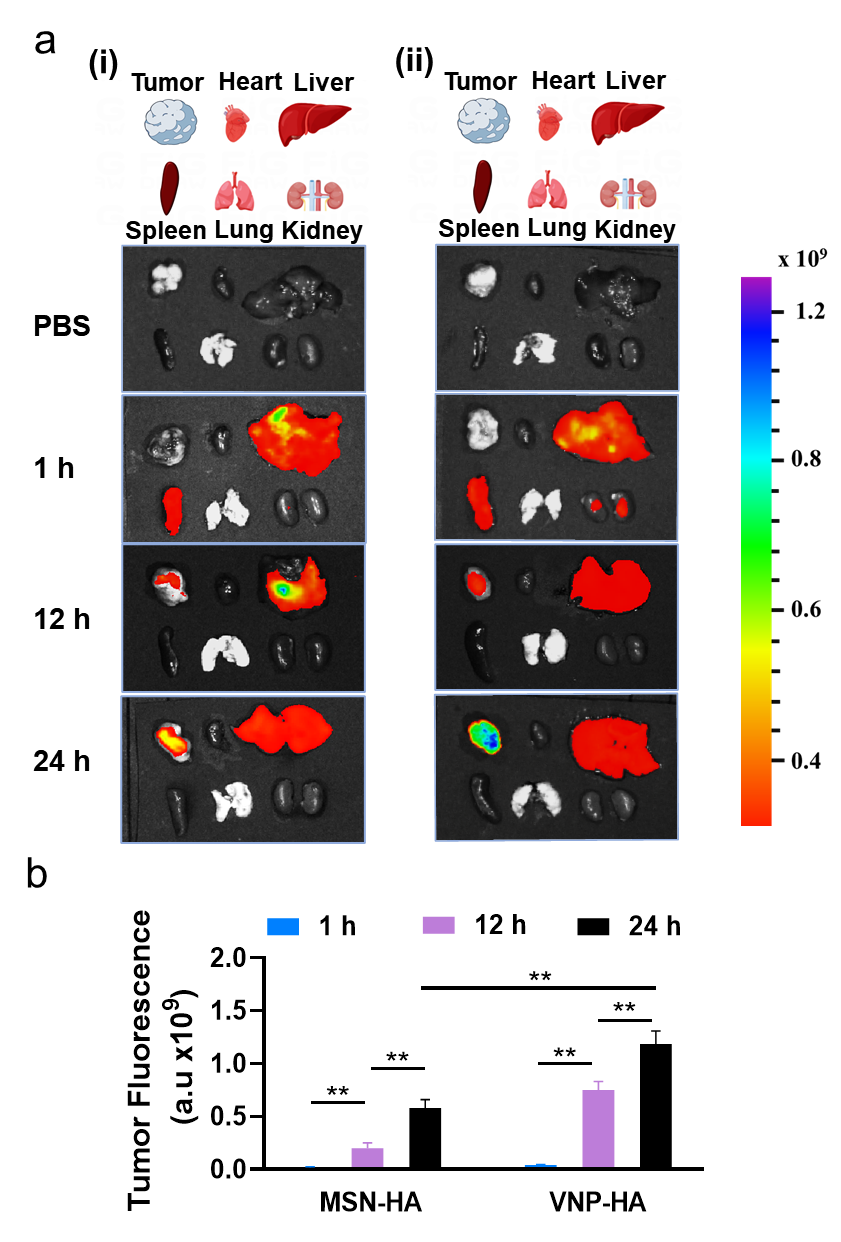


**Fig. S13.** (a) After the injection of (i) MSN-HA and (ii) VNP-HA in mice, ex vivo imaging of organs and tumors. (b) Their quantified accumulation in tumors at varying intervals. **P < 0.01 using one-way ANOVA.


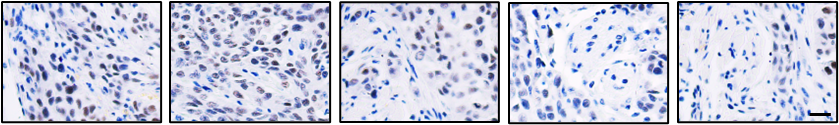


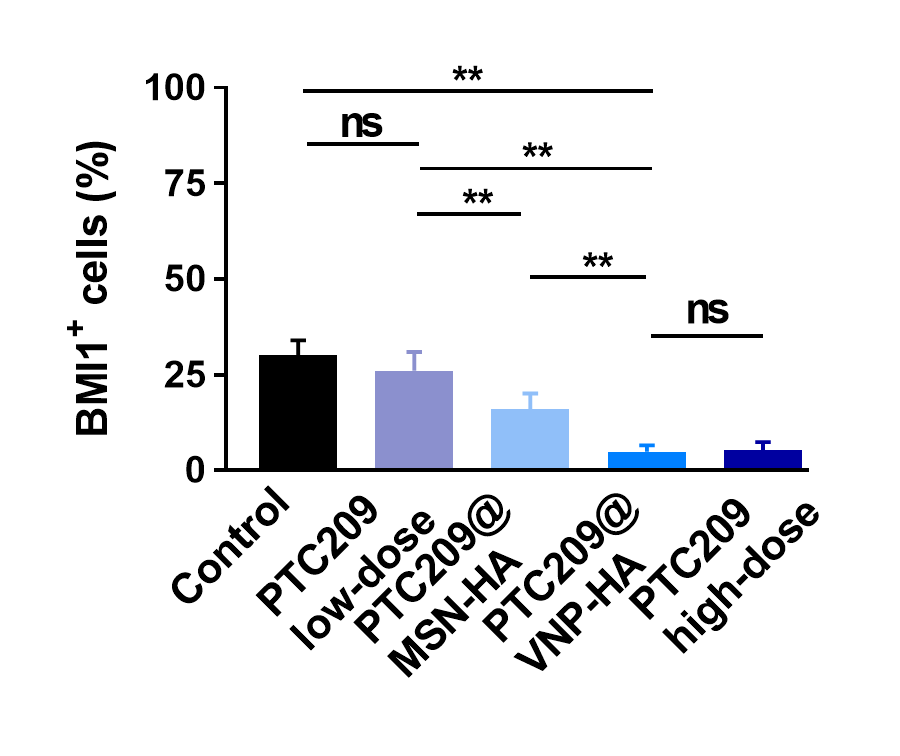


**Fig. S14.** Immunostaining and quantification of BMI1^+^ cells in tumors from orthotopic mice model with treatment as indicated. n = 6. Scale bar: 20 μm. Data are means ± SD. Asterisks indicate significant differences (**P < 0.01). Significance was evaluated using one-way ANOVA.


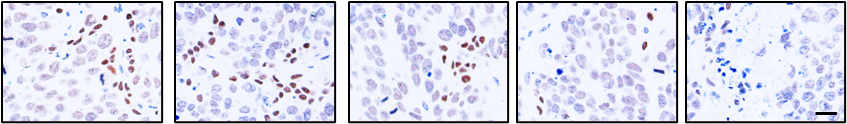


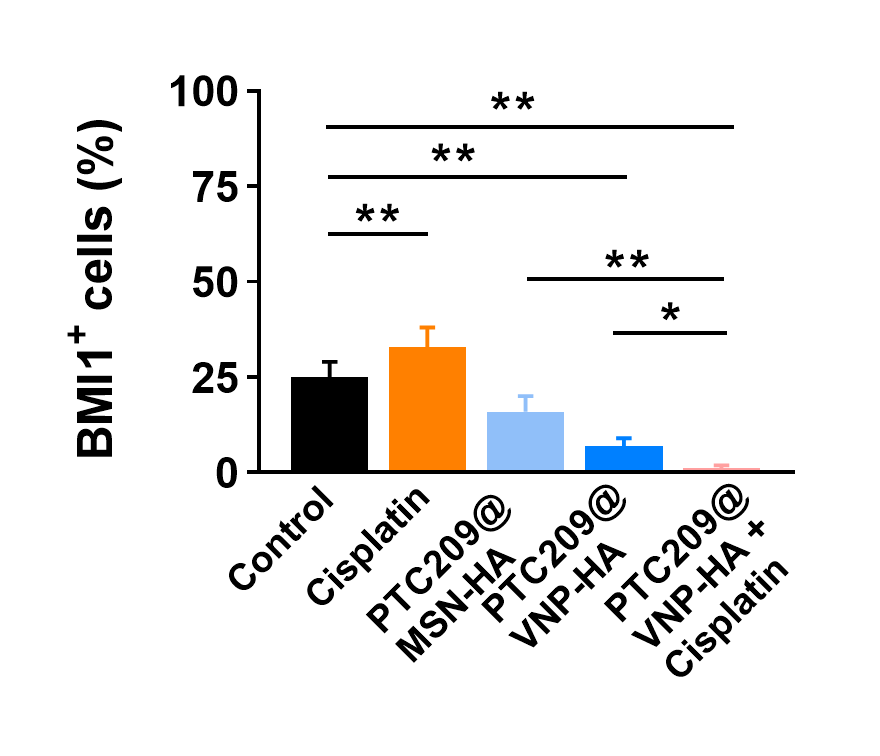


**Fig. S15.** Immunostaining and quantification of BMI1^+^ cells in tumors from in cisplatin resistance xenograft mouse model with treatment as indicated. n = 6. Scale bar: 20 μm. Data are means ± SD. Asterisks indicate significant differences (*P < 0.05, **P < 0.01). Significance was evaluated using one-way ANOVA.

**References**

[1] Y. Niu, M. Yu, J. Zhang, Y. Yang, C. Xu, M. Yeh, E. Taran, J.J.C. Hou, P.P. Gray, C. Yu, Synthesis of silica nanoparticles with controllable surface roughness for therapeutic protein delivery, Journal of Materials Chemistry B 3(43) (2015) 8477-8485. <https://doi.org/10.1039/c5tb01405k>.
